# Anti-*Candida albicans* effect of the protein-carbohydrate fraction obtained from the coelomic fluid of earthworm *Dendrobaena veneta*

**DOI:** 10.1371/journal.pone.0212869

**Published:** 2019-03-11

**Authors:** Marta J. Fiołka, Paulina Czaplewska, Katarzyna Macur, Tomasz Buchwald, Jolanta Kutkowska, Roman Paduch, Zbigniew Kaczyński, Jerzy Wydrych, Teresa Urbanik-Sypniewska

**Affiliations:** 1 Department of Immunobiology, Institute of Biology and Biochemistry, Maria Curie-Skłodowska University, Lublin, Poland; 2 Intercollegiate Faculty of Biotechnology UG-MUG Laboratory of Mass Spectrometry, Gdansk, Poland; 3 Institute of Material Research and Quantum Engineering, Faculty of Technical Physics, Poznań University of Technology, Poznań, Poland; 4 Department of Genetics and Microbiology, Faculty of Biology and Biotechnology Maria Curie-Skłodowska University, Lublin, Poland; 5 Department of Virology and Immunology, Institute of Microbiology and Biotechnology, Maria Curie-Skłodowska University, Lublin, Poland; 6 Department of Biomedical Chemistry, Laboratory of Structural Biochemistry, Faculty of Chemistry, University of Gdańsk, Gdańsk, Poland; 7 Department of Comparative Anatomy and Anthropology, Institute of Biology and Biochemistry, Maria Curie-Skłodowska University, Lublin, Poland; Tallinn University of Technology, ESTONIA

## Abstract

An antifungal active fraction (AAF) from the coelomic fluid (CF) of the earthworm *Dendrobaena veneta* was isolated. The aim of the study was to analyze the antifungal activity of the AAF and to carry out chemical characterization of the fraction. The active fraction showed antifungal activity against a clinical *C*. *albicans* isolate, *C*. *albicans* ATCC 10231, and *C*. *krusei* ATCC 6258. It effectively reduced the metabolic activity of *C*. *albicans* cells and influenced their morphology after 48 hours of incubation. Scanning electron microscopy (SEM) images revealed loss of integrity of the cell wall induced by the active fraction. Calcofluor White staining showed changes in the structure of the *C*. *albicans* cell wall induced by the AAF. The fungal cells died via apoptosis and necrosis after the treatment with the studied fraction. Electrophoresis under native conditions revealed the presence of two compounds in the AAF, while SDS/PAGE gel electrophoresis showed several protein and carbohydrate compounds. The active fraction was analyzed using Raman spectroscopy, MALDI TOF/TOF, and ESI LC-MS. The Raman analysis confirmed the presence of proteins and determined their secondary structure. The MALDI TOF/TOF analysis facilitated detection of four main compounds with a mass of 7694.9 m/z, 12292.3 m/z, 21628.3 m/z, and 42923.2 m/z in the analyzed fraction. The presence of carbohydrate compounds in the preparation was confirmed by nuclear magnetic resonance (NMR) and gas chromatography (GC-MS). The ATR-FTIR spectrum of the AAF exhibited high similarity to the spectrum of egg white lysozyme. The AAF showed no endotoxicity and cytotoxicity towards normal skin fibroblasts (HSF); therefore, it can be used for the treatment of skin and mucous membrane candidiasis in the future. Given its efficient and selective action, the fraction seems to be a promising preparation with antifungal activity against *C*. *albicans*.

## Introduction

*Candida albicans* is a commensal organism, which can be part of the normal microbiota in healthy humans. This *Ascomycete* yeast is able to cause diseases in individuals with a weakened and compromised immune system [1]. It can cause superficial or invasive infections with a high mortality rate. *Candida* species are responsible for various clinical manifestations, and *C*. *albicans* is the most common causative agent of diseases [2]. *Candida* spp. are reported as the cause of approximately 400,000 cases of systemic fungal diseases with mortality rates of up to 40% [3]. Therefore, despite the progress in medicine, candidiasis is still a serious clinical problem with a global range. In recent years, an increase in resistance to antibiotics in the treatment of fungal infections has been observed. This world-scale problem has created a need to search for new antifungal agents.

The medical potential of earthworms has been known worldwide for centuries. The use of pharmaceuticals derived from earthworms as green medicine is highly developed in China and other Asian countries [4]. Extracts prepared from tissues of these invertebrates are used to treat many diseases. Many molecules derived from earthworm tissue extracts exhibit antimicrobial, anti-inflammatory, and anti-tumor activity [5]. New research is still being undertaken to indicate the possibility of a clinical use of earthworm components [6].

There are few reports describing the antifungal activity of earthworm tissues [7–10]. Most of these publications describe antifungal activity in extracts prepared as pastes and powders from whole earthworms. Others describe the activity of one antimicrobial peptide isolated from two species of earthworms [11, 12] The antifungal activity of metabolites produced by symbiotic bacteria associated with the earthworm gut has been characterized as well [13–15].

Studies conducted by us have proved that earthworms *Dendrobaena veneta* have the potential to fight *C*. *albicans* fungi, and the *D*. *veneta* gut bacterium–*Raoultella ornithinolytica* has the ability to produce extracellular metabolites with antifungal and anticancer properties [14, 15]. The coelomic fluid (CF) of this earthworm species has not been tested in this respect yet; therefore, it seemed reasonable to carry out such analyses.

The results of earlier research have indicated that the earthworm CF contains bioactive compounds such as antibacterial peptides [16], metalloenzymes [17], fibrinolytic enzymes [18], proteases [19], lectins [20], polysaccharides [21], etc. The biological activities were mainly assigned to proteins and peptides isolated from earthworms [22–25]. Cytotoxicity to vertebrate cells is the most undesirable property among the various activities of the CF [26].

The aim of this study was to prepare a non-toxic protein extract from the earthworm CF and examine its activity against *C*. *albicans*. To date, there have been no reports on an antifungal preparation derived from earthworm CF that does not exhibit cytotoxic activity against normal somatic cells.

## Material and methods

### Earthworms

Mature earthworms *Dendrobaena veneta* were maintained in the laboratory culture of the Department of Immunobiology, Maria Curie Skłodowska University in Lublin. The animals were kept in containers filled with compost soil at ca. 20°C in the dark. The animals were fed with boiled vegetables twice a week. Adult earthworms were chosen for the experiments.

### Harvesting of the earthworm CF

After intense cleaning of earthworm guts for 24 h, the CF was harvested by mild electrical stimulation (4.5V) from groups of 10 pooled earthworms. The CF containing coelomocytes was collected in 0.9% NaCl (1500 μl per group). Then, the CF was centrifuged at 6.000 rpm for 10 min at 4°C to separate it from the coelomocytes. The cell-free supernatant was sterilized by filtration through 0.22 μm Millipore filters. The cell-free CF was heated for 10 min at 70°C, transferred to a cellulose membrane bag with cut off points of 12–14 kDa, and dialyzed against water for 24 h at 4°C. The fraction obtained (AAF) after the dialysis was pipetted into Eppendorf tubes and lyophilized. The samples designed for the experiments were stored at -20°C. The protein concentration was estimated using the Bradford assay (Bio-Rad) [27]. Approximately 1 mg of AAF protein was obtained from fifteen adult earthworms. Subsequently, ultrafiltration with a centrifugal filter device Amicon Ultra-4 (Millipore) with cut off points of 30 and 100 kDa was performed. Subfractions containing compounds of 14–30 kDa, 30–100 kDa, and above 100 kDa were obtained. Both the unseparated fraction and the subfractions were used for further analysis.

### Microorganisms and preparation for microscopy

The action of AAF on *Candida* strains: *C*. *albicans*–a wild-type clinical isolate (kindly gifted by Prof. A Kędzia, Department of Oral Microbiology, Medical University of Gdańsk), *C*. *albicans* ATCC 10231, and *C*. *krusei* (another name *Issatchenkia orientalis*) ATCC 6258 were grown in a liquid YPD medium (1% yeast extract, 2% peptone, 2% dextrose) at 28°C.

The AAF at the final concentrations of 6.25, 12.5, 25, 100, and 200 μg mL^-1^ was added to 150 μL YPD liquid poor medium (15) containing the *Candida* culture (10^7^ CFU from the logarithmic phase in YPD). Then, the suspension was supplemented with streptomycin sulfate (Sigma) (0.17 mg mL^-1^) and completed with YPD poor medium to the final volume of 250 μL. The samples were incubated for 48 hours at 37°C with gentle shaking. After that time, the antifungal activity of the AAF was analyzed under fluorescent and scanning electron microscopes.

### Metabolic activity of *C*. *albicans* cells after treatment with the AAF

The activity of the AAF at the protein concentration of 6.25, 12.5, 25, 50, 100, and 200 μg mL^-1^ was analyzed. The determination of the metabolic activity of the three *Candida* strains was carried out using the LIVE/DEAD Yeast Viability Kit F-7030 FUN 1 [28].

After incubation with the AAF, the *C*. *albicans* or *C*. *krusei* cell suspension was centrifuged for 5 min at 10 000 rpm at room temperature, and the fungal cells were resuspended in 100 μL of the GH solution (2% D-glucose containing 10 mmol L^-1^ Na-HEPES, pH 7.2). 30 μL of the *Candida* suspension in the GH solution were added to 30 μL of the GH-FUN solution. The samples were incubated for 30 min at 30°C in the dark before microscopic analysis.

Fluorescent staining for quantification of the viability of the *C*. *albicans* cells was applied as described by Fiołka et al. [14]. Only metabolically active cells were clearly marked with fluorescent intravacuolar red structures. Dead cells exhibited extremely bright, diffuse, green-yellow fluorescence. Cells with intact membranes and showing little or no metabolic activity exhibited diffused green cytoplasmic fluorescence and had no fluorescent intravacuolar bodies.

The minimum inhibitory concentrations (MICs) for the three strains of *C*. *albicans*, i.e. the clinical isolate, *C*. *albicans* ATCC 10231, and *C*. *krusei* ATCC 6258, were determined with the broth microdilution method as specified by the Clinical and Laboratory Standards Institute (CLSI) [29]. The AAF with protein concentrations ranging from 3.125 to 50 mg mL^-1^ was analyzed. Cell growth was measured using a Benchmark Plus spectrophotometer at 600 nm (Bio-Rad). The experiment was repeated three times.

The wild-type clinical isolate of *C*. *albicans* was used for further microscopic analysis.

### Imaging cell morphology after Calcofluor White staining

The *C*. *albicans* control culture and the culture incubated with the AAF were stained with Calcofluor White (Fluka), which binds to chitin in the fungal cell wall [30]. To prepare the fluorochrome solution, 3 μl of the dye were added to 15 μl of sterile water. Next, 10 μl of the solution with 10 μl of *C*. *albicans* cell suspension were mixed in a separate Eppendorf tube. The mixture was incubated for 10 minutes at room temperature in the dark. Then, 2 mL of the mixture was dropped onto a slide and images of *Candida* cells were documented with a microscope. The fungal cells were observed at 1000 x magnification and imaged using a Zeiss/LEO 912AB microscope.

### Scanning electron microscopy (SEM) analysis

The standard SEM procedure involved fixation of the fungal cells with 4% glutaraldehyde in 0.1 M phosphate buffer, pH 7.0, treatment with OsO_4_, and dehydration in a graded acetone series (in 15%, 30%, 50%, 70%, and 100% for 30 min for each concentration, at 3000 rpm). The preparations were dried in a desiccator using silica gel beads for 24 hours and then gold sputtered using a K550X sputter coater (Quorum Technologies). A Vega 3 scanning electron microscope (Tescan) was used for examination of the samples.

The AAF structure was observed and documented using a scanning electron microscope Quanta 3D FEG. The lyophilized preparation of the AAF was used for the observation. It was not subjected to standard dehydration and gold sputtering, which allowed visualization of its morphology in the most natural shape.

### Identification of apoptotic and necrotic *C*. *albicans* cells

For identification of apoptosis and necrosis, the *C*. *albicans* cells were stained with a mixture of fluorescent Hoechst 33342 (Sigma) and propidium iodide (Sigma) dyes. The cells were visualized with a Zeiss LSM 5 Pascal fluorescence microscope at excitation and emission wavelengths of ~460 nm and >575 nm. The staining mixture was added to the culture (2.5 μl/mL^-1^) and incubated for 5 min at 37°C in the dark. Cells undergoing apoptosis had intact or fragmented blue fluorescent nuclei. Cells with pink fluorescent nuclei were considered necrotic.

### Fourier transform-infrared spectroscopy (FTIR) analysis of the AAF

A FTIR microscope iN10MX (Thermo Scientific, Waltham, MA, USA) was used for the analysis. Molecular structures were identified by infrared absorption bands. The spectra obtained from five analyzed areas (50 x 50 μm) of the AAF were compiled and compared with each other. The analyses were carried out at room temperature directly on the sample surface using the reflection technique at the spectral range 650–4000 cm^-1^ with a resolution of 4 cm^-1^.

The FTIR spectra of the AAF and egg white lysozyme (Fluka) were compared using a FTIR Nicolet 8700A spectrometer (Thermo Scientific, Waltham, MA, USA). The analyses were carried out at room temperature directly on the surface of the sample by ATR with a diamond crystal at a spectral range of 400–4000 cm^-1^ with a resolution of 4 cm^-1^.

### Lysozyme activity of the AAF

The activity of the AAF against the Gram-positive indicator bacterium *Micrococcus luteus* was determined with a spectrophotometric assay [31] applied to freeze-dried cell walls of *M*. *luteus* (Sigma) using a suspension of dead cells (1 mg mL^-1^) in 33 mM phosphate buffer pH 6.8. The rate of cell wall hydrolysis was measured at 450 nm with the use of a Bio-Rad spectrophotometer after 15-s incubation at 25°C. The lysozyme activity was calculated in Units/mg. A unit of activity was defined as a change in absorbance of 0.001 per 1 min in these conditions. The protein concentration was estimated using the Bradford method [27].

### Polyacrylamide gel electrophoresis analysis

SDS–polyacrylamide gel electrophoresis (SDS–PAGE) was performed with the Laemmli method [32] in 10% acrylamide gels. The AAF (20 μg of protein) samples were heated at 100°C for 3 min in the sample buffer. Protein bands were detected by staining with Coomassie Brilliant Blue R-250 (Sigma). Molecular weight markers (Bio-Rad) (SM0431) were used. To detect carbohydrate compounds, the gels were stained with silver nitrate after sodium periodate oxidation [33].

The AAF was analyzed with analytical electrophoresis according to Reisfeld et al. [34] in 15% polyacrylamide gel at pH 4.3 for 1 h at 150 V. The lysozyme-type compounds were visualized in gel using bioautography as an activity assay. The gel was washed in 33 mM Sörensen buffer, pH 6.4 for 30 min. Next, the gel was overlaid with 10 ml of 33 mM Sörensen buffer containing 10 mg freeze-dried cells of *M*. *luteus* in 1% agarose. Lysozyme-type protein bands with bacteriolytic activities were identified from clearance zones in the bacterium-containing agarose overlay after 48 h incubation at 30°C. The bioautography assay was repeated three times.

### MALDI analysis of the AAF

The MS measurements of the intact AAF were carried out with the use of MALDI TOF/TOF 5800 (ABSciex, Framingham, MA, USA). Sinapinic acid (SA, Sigma-Aldrich), 2,5-dihydroxybenzoic acid (DHB, Sigma-Aldrich), and a binary matrix superDHB (DHB and 2-hydroxy-5-methoxybenzoic acid mixture, sDHB, Sigma-Aldrich) were used as matrices. In all cases, the concentration of the matrices was 10 μg mL^-1^. They were dissolved in 50% acetonitrile (ACN), 50% water, and 0.1% trifluoroacetic acid (TFA). The measurements were performed in positive ion linear middle and high mass modes with previous mass calibration with a commercial mAb Mass Check Standard (Waters, Milford, MA, USA) and CalMix 3 (ABSciex, Germany). The lyophilized AAF sample was dissolved in 1 ml of water (MS grade) to a final concentration of 1 mg mL^-1^ and sonicated for 10 min in 40°C. MALDI spots were prepared with the dried droplet preparation method by mixing 0.6 μl of an AAF solution with 0.6 μl of a matrix solution (directly on the plate). After air-drying, the plate was introduced directly to the instrument. MS spectra were acquired from 5 000 to 50 000 m/z (linear middle mass mode) and from 30 000 to 90 000 m/z (linear high mass mode) for a total of 1000 laser shots by an 1 kHz OptiBeam laser (YAG, 349 nm). The laser intensity was fixed for all the analyses. The registered spectra were analyzed with Data Explorer software (ABSciex).

### Sample preparation for LC-MS/MS analysis

In-solution digestion: the AAF sample was dissolved in 8 M urea in 50 mM amonium bicarbonate (NH_4_HCO_3_) to a final concentration of 30 μg μL^-1^. First, reduction and alkylation were performed (50 mM dithiothreitol, 150 mM iodoacetamide IAA in 50 mM NH_4_HCO_3_). The reduced and alkylated sample was divided into three vials and digested overnight individually with trypsin, chymotrypsin, and endopeptidase Lys-C, E:S 1:50 w/w [35]. The digestion was stopped by acidifying and desalting the solution according to the standard protocol [36].

In-gel digestion: Cuts from the 2D gel bands were digested with trypsin according to the standard protocol [37].

#### LC-MS/MS analysis

After desalting, the samples were analyzed in a positive ion mode on a TripleTOF 5600+ hybrid mass spectrometer with a DuoSpray Ion Source (AB SCIEX, Framingham, MA, USA) connected with an Ekspert MicroLC 200 Plus System (Eksigent, Redwood City, CA, USA). Column parameters: Exigent microLC column ChromXP C18CL (3 μm, 120 Å, 150 × 0.3 mm). The samples were loaded onto the column using a CTC Pal Autosampler (CTC Analytics AG, Zwinger, Switzerland) at an injection volume of 5 μl. Solvents A and B consisted of 0.1% (v/v) formic acid (FA) in water and acetonitrile, respectively. LC gradient parameters: 10–90% solvent B in 28 min, flow rate of 10 μl/min, followed by 2-min regeneration and re-equilibration. The microLC-MS/MS system was controlled by the AB SCIEX Analyst TF 1.6 software. The data-dependent experiments (IDA) were conducted for all the investigated samples as described previously [36].

#### Data analysis

The protein database searches were conducted in ProteinPilot (software ver 4.5, AB SCIEX) using the Paragon algorithm against the Uniprot databases (ver. 8.11.2017, Annelida, Lophotrochozoa) with an automated false discovery rate (FDR). Search parameters: instrument TripleTOF 5600, alkylation of the cysteine residue by iodoacetamide, trypsin/chymotrypsin/Lys-C enzyme digestion, ID focus on biological modifications, search effort “thorough ID” and detected protein threshold [Conf] > 10%, and peptide confidence ≥ 95% and peptide FDR < 1% for identification of peptides.

### Raman spectroscopy of the AAF

The secondary structure of the AAF proteins was analyzed using Raman spectroscopy. The measurements were carried out with an inVia Renishaw microscope equipped with a diode-pumped laser emitting a 785 nm wavelength. During the measurements, the laser beam was automatically focused on the sample through a Leica 100 x microscope objective with a numerical aperture of 0.85, yielding a laser beam diameter of approx. 1.1 μm. An air-cooled CCD Camera detector (Rencam) and 1200 l/mm diffraction grating were used. At the beginning of each experimental session, the spectral data were regularly calibrated with the use of the 520.7 cm^-1^ Raman band of a silicon internal reference sample. Twenty Raman spectra of proteins were recorded in the backscattering geometry in the spectral range from 200 cm^-1^ to 3200 cm^-1^. The time of light exposure to get single Raman spectra was 10 s and the spectra were recorded with 10 accumulations.

The samples were studied at room temperature (about 23°C). The analyses of the spectra were performed using WiRE 3.4 (Renishaw) software. The background in the Raman scattering spectrum was subtracted using a polynomial curve. The integrated intensity of the Raman bands was used to detect the quantity of the secondary structures in the studied material. The integrated intensity of bands associated with random coil, alpha helix, beta sheet, and beta turn structures were calculated by the curve-fitting process in a range of the amide I band from 1590 cm^-1^ to 1710 cm^-1^.

### NMR analysis of the AAF

^1^H NMR (nuclear magnetic resonance) spectra were recorded at 25°C using a Bruker Avance III 500 MHz spectrometer. The samples (~2 mg) were dissolved in 0.75 mL of 99.9% D_2_O. The spectra were referenced to acetone (δ_H_ 2.22).

### Carbohydrate composition of the AAF

The carbohydrate content of the AAF fraction was determined as alditol acetates. The acid-hydrolyzed samples (2 M TFA, 120°C, 2 h) were converted into alditol acetate derivatives with the procedure proposed by Albersheim et al. [38]. The GC-MS analysis was performed using a 7890A Agilent gas chromatograph (Agilent Technologies, Wilmington, DE, USA) connected to a mass selective detector (5975C XL EI/CI) equipped with a capillary column (HP-5MS, 30 m x 0.25 mm) with helium as a carrier gas. The GC oven temperature program was as follows: the initial oven temperature was set at 150°C and held for 5 min; next, the temperature was increased to 310°C via a ramp of 5°C min^−1^ and maintained for 10 min.

Data acquisition and integration were carried out with the HPCHEM chromatography software. The components were identified by comparison of their mass spectra with those of standards and the spectrometer database using the NIST library (National Institute of Standards and Technology, Gaithersburg, MD, USA), with probabilities higher than 80%.

### Determination of cytotoxicity towards HSF

#### Establishment of the HSF cell culture

Freshly excised fragments of human skin were washed twice using RPMI 1640 medium (Gibco, Paisley, UK) supplemented with antibiotics (100 U mL^-1^ penicillin, 100 μg mL^-1^ streptomycin, 0.25 μg mL^-1^ amphotericin B) (Gibco) and then placed into the wells of a 24-well plate. The explants were then overlaid with a warm 1:1 (v/v) mixture of 1% agarose and RPMI 1640 medium. The culture was performed by adding culture medium RPMI 1640 supplemented with 10% fetal bovine serum (FBS) (Gibco) on agarose gel and incubated at 37°C in a humidified air incubator (5% CO_2_/95% air). Outgrowths of skin fibroblasts were separated and cultured. HSF cells obtained from two donors were used in the experiments.

#### Skin fibroblast culture

The HSF cells were cultured as monolayers in 25 cm^2^ culture flasks (Nunc. Roskilde, Denmark) in RPMI 1640 medium supplemented with 10% FBS (v/v) and antibiotics at 37°C in a humidified atmosphere with 5% CO_2_. The total number of cells used in the experiments was counted in a hemocytometer. 100 μl of cell suspension (1 × 10^5^ cells/ml) was added to appropriate wells of 96-well flat-bottomed microtiter plates. After 24 h of incubation, the medium was discarded and new media containing 2% FBS and appropriate concentrations of the AAF (12.5–200 μg mL^-1^ range) were added. The HSF cells suspended in 100 μl of culture medium with 2% FBS without AAF addition were used as a control. The total number of cells was equivalent to that in the sample wells.

#### MTT assay

The sensitivity of the cells to the AAF activity was determined with a standard spectrophotometric 3-(4,5-dimethylthiazole-2-yl)-2,5-diphenyltetrazolium bromide (MTT) assay. Cells grown in 96-well multiplates in 100 μl of culture medium supplemented with 2% FBS were incubated for 3 h with an MTT solution (5 mg mL^-1^, 25 μl/well) (Sigma, St. Louis, MO, USA). The yellow tetrazolium salt was metabolized by viable cells to purple formazan crystals. The crystals were solubilized overnight in a mixture of 10% sodium dodecyl sulfate (SDS) (Sigma) in 0.01 M HCl. The product was quantified spectrophotometrically by measuring the absorbance at a 570 nm wavelength using an E-max Microplate Reader (Molecular Devices Corporation, Menlo Park, CA, USA).

#### Staining of argyrophilic nucleolar organizer regions (AgNORs)

After incubation in 4-well Lab-Tek II Chamber slides and 24-h incubation with AAF, the cells were rinsed with PBS and fixed with an absolute ethanol/acetic acid solution (1:1) for 10 min. The silver colloid solution was prepared using 2% gelatin in 1% formic acid mixed with 30% aqueous silver nitrate at 1:2 volumes. The cells were immersed in this solution for 5 min at 37°C, rinsed with deionized water, and mounted with glycerol. AgNOR proteins were determined using a computer-assisted image analysis system.

### Endotoxin content in the AAF

The content of endotoxin in the high-molecular fraction of the CF was determined with a LAL Chromogenic Endotoxin Quantitation Kit (Thermo Scientific, Pierce) according to the manufacturer's protocol. The endotoxin concentration was expressed as endotoxin units (EU) using *E*. *coli* O111:B4 lipopolysaccharide to perform a calibration curve.

### Effect of temperature and pH on anti-*C*. *albicans* activity

After dissolution in sterile deionized water (pH 6.5), AAF was incubated for 10 min at 25°C, 45°C, 65°C, and 85°C and then added to the medium with *C*. *albicans* ATCC 10231 cells to the final protein concentration of 100 μg/ml. Incubation was carried out at 37°C for 48 hours and then the metabolic activity of *C*. *albicans* was determined as described in section 2.4. AAF was also dissolved in Sörensen buffer at pH 5.0, 5.5, 6, 6.5, 7, and 7.5 and incubated for 10 min at room temperature, and the experiment was carried out as described above.

### Statistical analysis

Statistical analysis was performed using the one way ANOVA with Dunnett’s multiple comparison test. The results were expressed as mean ±SD (standard deviation). The analysis of the metabolic activity of fungal cells and skin fibroblasts was performed for data from three independent experiments.

## Results

### Metabolic activity of *C*. *albicans* cells after treatment with the AAF

**Fig 1:**
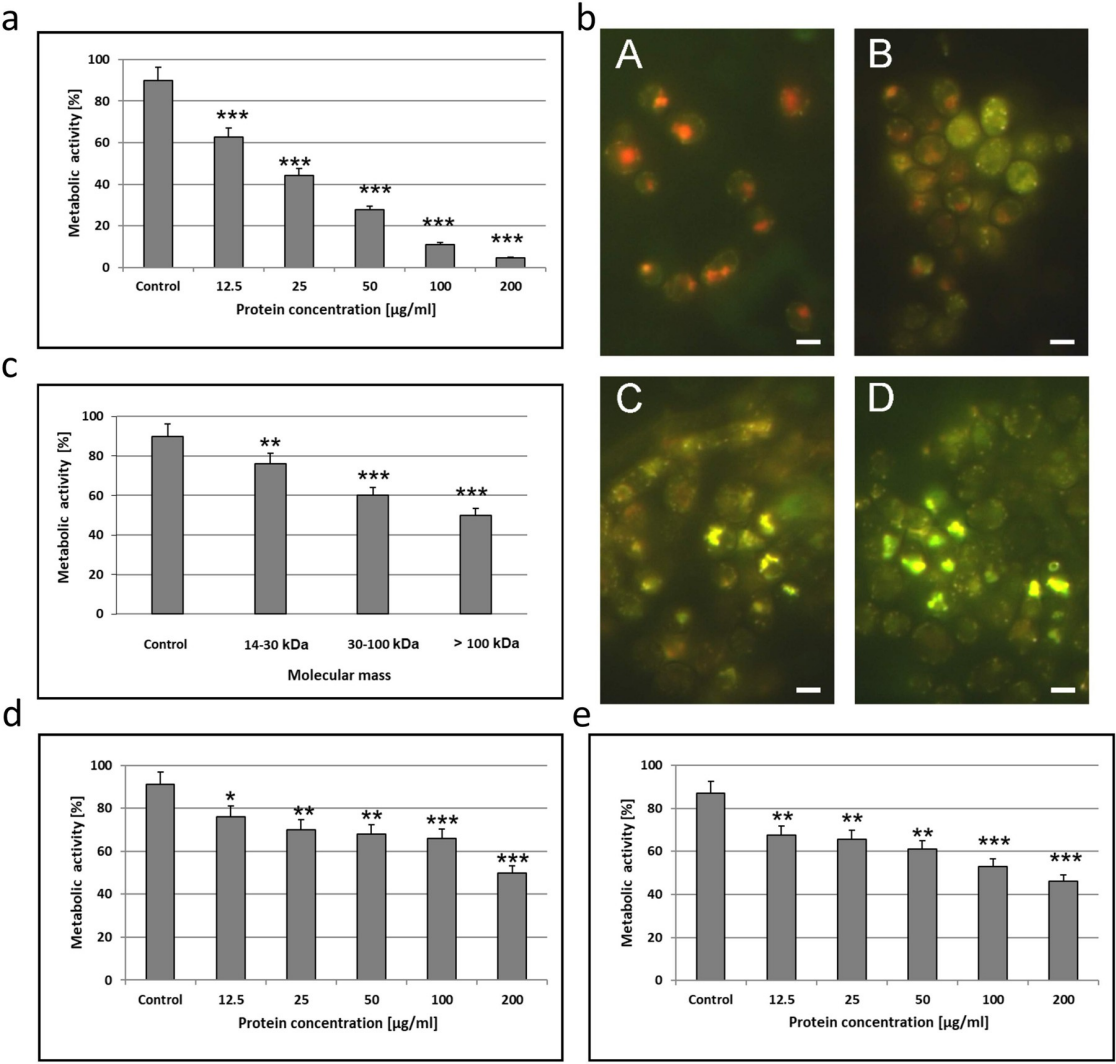
**a)** Metabolic activity of *C*. *albicans* cells (clinical isolate) after incubation for 48 h with the AAF at different protein concentrations. The data are representative of three independent experiments, *p<0.001; **b)** Metabolic activity of *C*. *albicans* cells (clinical isolate) after incubation for 48 h with the AAF observed under the fluorescence microscope; A—*C*. *albicans* cells, control culture–(metabolically active cells were clearly marked with fluorescent intravacuolar red structures), B—*C*. *albicans* cells after treatment with the AAF at the concentration of 25 μg mL^-1^ (cells with intact membranes showing low or no metabolic activity exhibited diffused green cytoplasmic fluorescence), C—at the concentration of 50 μg mL^-1^, D—at the concentration of 100 μg mL^-1^ (dead cells exhibited extremely bright, diffuse, green-yellow fluorescence and absence of fluorescent intravacuolar bodies). Bars represent 2 μm; **c)** Metabolic activity of *C*. *albicans* cells (clinical isolate) after incubation for 48 h with different molecular mass subfractions at the protein concentration of 100 μg mL^-1^, **d)** Metabolic activity of *C*. *albicans* ATCC 10231 and **e)** *C*. *krusei* 6258 after incubation for 48 h with the AAF at different protein concentrations. The results were obtained from 3 independent experiments; ***P <0.001, **P<0.01, *P<0.05 compared to the control group.

The active fraction induced a significant decrease in the metabolic activity of the wild type *C*. *albicans* cells, even after the incubation at the low concentrations of protein. The metabolic activity of the control culture cells was 90% after 48 hours and 62.5% after the incubation with the AAF at the dose of 12.5 μg mL^-1^. The metabolic activity was 44.3% after the application of the AAF with a protein concentration of 25 μg mL^-1^ and 27.6% at the protein concentration of 50 μg mL^-1^. At the AAF concentrations of 100 μg mL^-1^ and 200 μg mL^-1^, the activity was 11.1% and 4.7%, respectively (Fig 1A). In each case, the differences were statistically significant.

Fig 1B shows *C*. *abicans* cells visualized with the use of the fluorescence microscope after incubation with the different concentrations of AAF. There was a visible change in the color of the *C*. *albicans* cells. The number of living cells with red inclusions decreased, while the number of green fluorescing (metabolically inactive) cells and yellow fluorescing (dead) cells increased with the increasing concentration of the AAF.

The metabolic activity of the wild-type *C*. *albicans* cells after the incubation with the fraction (at the concentration of 100 μg mL^-1^) containing compounds with a molecular mass of 14–30 kDa was 76% (24% activity reduction, compared to the control). In the presence of compounds with a molecular mass of 30–100 kDa and above 100 kDa, the metabolic activity was 60% (30% reduction) and 50% (40% reduction), respectively (Fig 1C). Each separated fraction exhibited weaker antifungal metabolic activity than the AAF.

The MIC for all analyzed strains: the clinical *C*. *albicans* isolate, *C*. *albicans* ATCC 10231, and *C*. *krusei* ATCC 6258 was 12.5 μg mL^-1^. The last two *Candida* strains were less sensitive to the AAF although the MIC was the same. At higher concentrations, the AAF resulted in lower reduction of the metabolic activity than in the case of the clinical *C*. *albicans* isolate (Fig 1D and 1E).

### Imaging cell morphology after Calcofluor White staining

**Fig 2:**
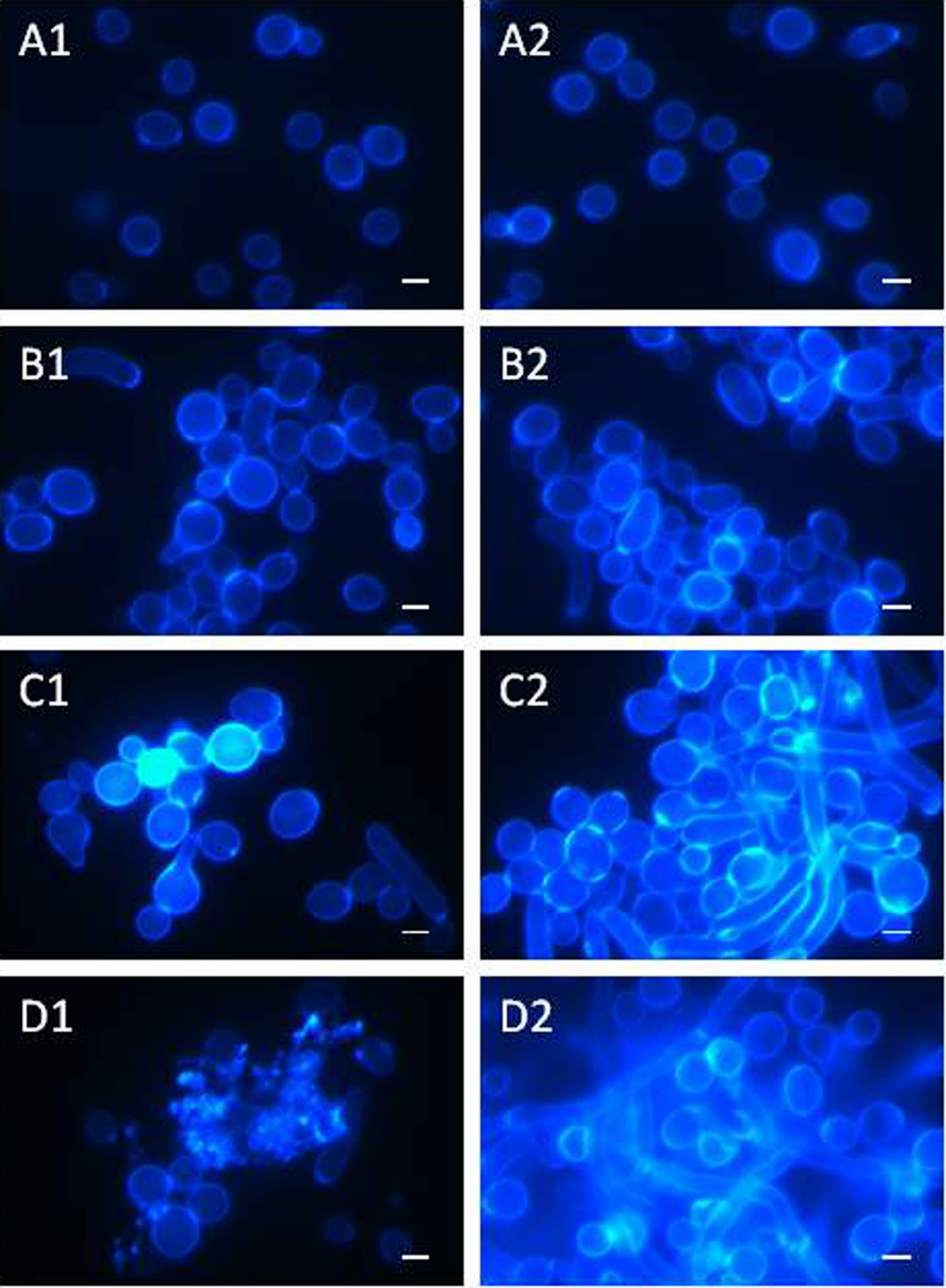
Morphological and cell structure changes in *C*. *albicans* (clinical isolate) after incubation with the AAF for 48 h at the different protein concentrations observed under the CLSM microscope after Calcofluor White staining; A1, A2—*C*. *albicans* control cells, B1, B2- *C*. *albicans* after incubation with the AAF at the concentration of 25 μg mL^-1^; C1, C2,- at the concentration of 50 μg mL^-1^, D1, D2—at the concentration of 100 μg mL^-1^. Bars represent 2 μm.

After the staining of the clinical *C*. *albicans* isolate with the Calcofluor White fluorochrome, the control cells had a regular shape and occurred singly (Fig 2A1 and 2A2). After the incubation with the AAF at the concentration of 25 μg mL^-1^, there was a tendency towards aggregation of several or more cells into groups, and some cells had an elongated shape (Fig 2B1 and 2B2). After the application of the AAF at a concentration of 50 μg mL^-1^, the cellular aggregates were larger and the number of irregularly shaped cells increased (Fig 2C1 and 2C2). The cell wall thickenings observed frequently in the agglomerates were characterized by intensive fluorescence. The AAF at a concentration of 100 μg mL^-1^ contributed to disintegration of the cells, which is shown in Fig 2D1. Disintegrated cells emitted blue fluorescence in the microscopic image, as shown in Fig 2D2.

### Scanning electron microscopy (SEM) analysis of *C*. *albicans* cells after incubation with the AAF

**Fig 3:**
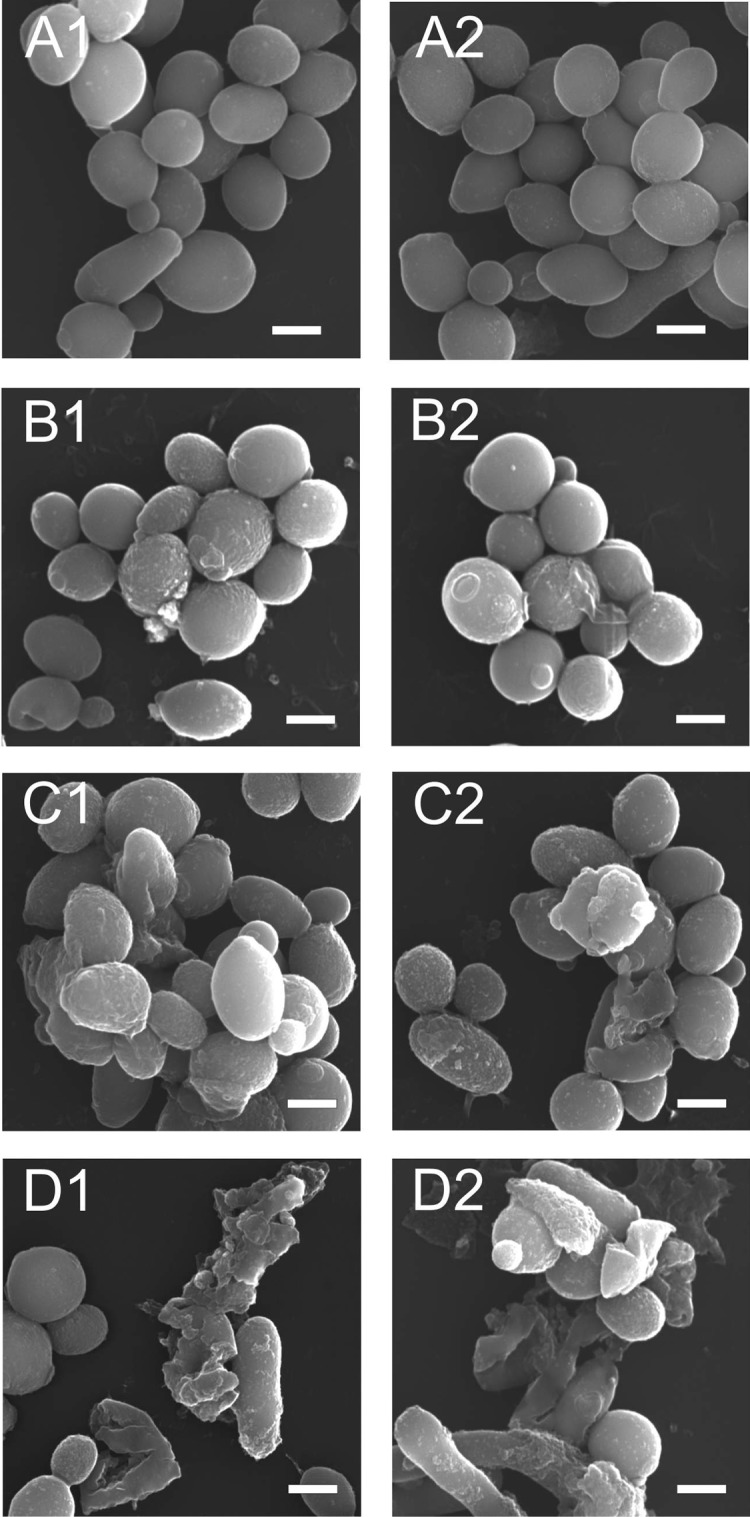
SEM image of the clinical *C*. *albicans* isolate after the incubation with the AAF for 48 h at the different protein concentrations, A1, A2- *C*. *albicans* control cells, B1, B2- *C*. *albicans* after the incubation with the AAF at the concentration of 25 μg mL^-1^,C1, C2,—at the concentration of 50 μg mL^-1^, D1, D2—at the concentration of 100 μg mL^-1^. Bars represent 2 μm.

*C*. *albicans* cells (clinical isolate) were incubated with the active fraction at various concentrations. Next, the cells were fixed and observed under a scanning electron microscope. The control cells were characterized by a regular shape with a smooth cell wall (Fig 2A1 and 2A2). There was a tendency to form aggregates composed of a dozen or so cells after the incubation with the AAF at a concentration of 25 μg mL^-1^ (Fig 3B1 and 3B2). Cells present in the middle of the clusters had a distinctly rough cell wall. After the addition of the fraction at 50 μg mL^-1^, the cells lost the integrity of the cell wall and their regular shape (Fig 2B1 and 2B2). After the incubation of the *C*. *albicans* cells with the AAF at a concentration of 100 μg mL^-1^, few cells retained their natural form. Most of the visible objects were either disintegrating cells or cellular fractions of already disintegrated cells (Fig 3D1 and 3D2).

### Identification of apoptotic and necrotic *C*. *albicans* cells

**Fig 4:**
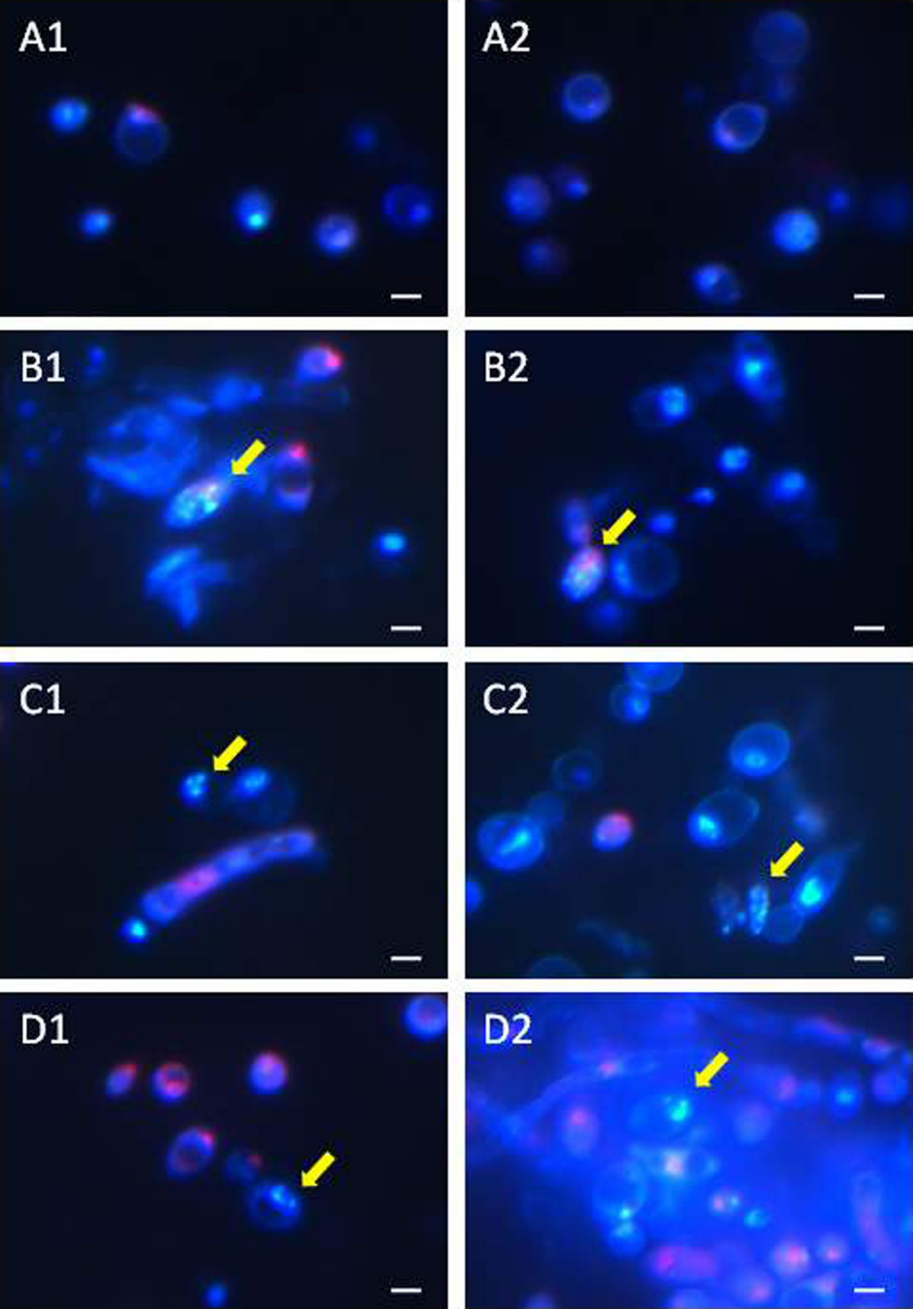
Apoptotic and necrotic *C*. *albicans* cells (clinical isolate) after the incubation with the AAF for 48 h at the different protein concentrations, A1, A2—*C*. *albicans* control cells, B1, B2—*C*. *albicans* after the incubation with the AAF at the concentration of 25 μg mL^-1^, C1, C2,—at the concentration of 50 μg mL^-1^, D1, D2—at the concentration of 100 μg mL^-1^. Normal cells are stained blue, necrotic cells are stained pink, and apoptotic cells have blue intact or fragmented fluorescent nuclei (indicated by arrows). Bars represent 2 μm.

After the staining of the AAF-treated *C*. *albicans* cells (clinical isolate) for identification of the cell death pathway, the cells treated with the fractions died via either apoptosis or necrosis (Fig 4). Fluorescence microscopy revealed pink fluorescing necrotic cells and apoptotic cells with a clearly fragmented brightly fluorescing cell nucleus (marked with arrows) after the incubation with the AAF at each concentration, i.e. 25 μg mL^-1^, 50 μg mL^-1^, and 100 μg mL^-1^. Additionally, after the incubation at a concentration of 100 μg mL^-1^, blue fluorescence was emitted by the disintegrated cells, which was visible in the microscope image (Fig 4D2).

### SEM analysis of the AAF

**Fig 5:**
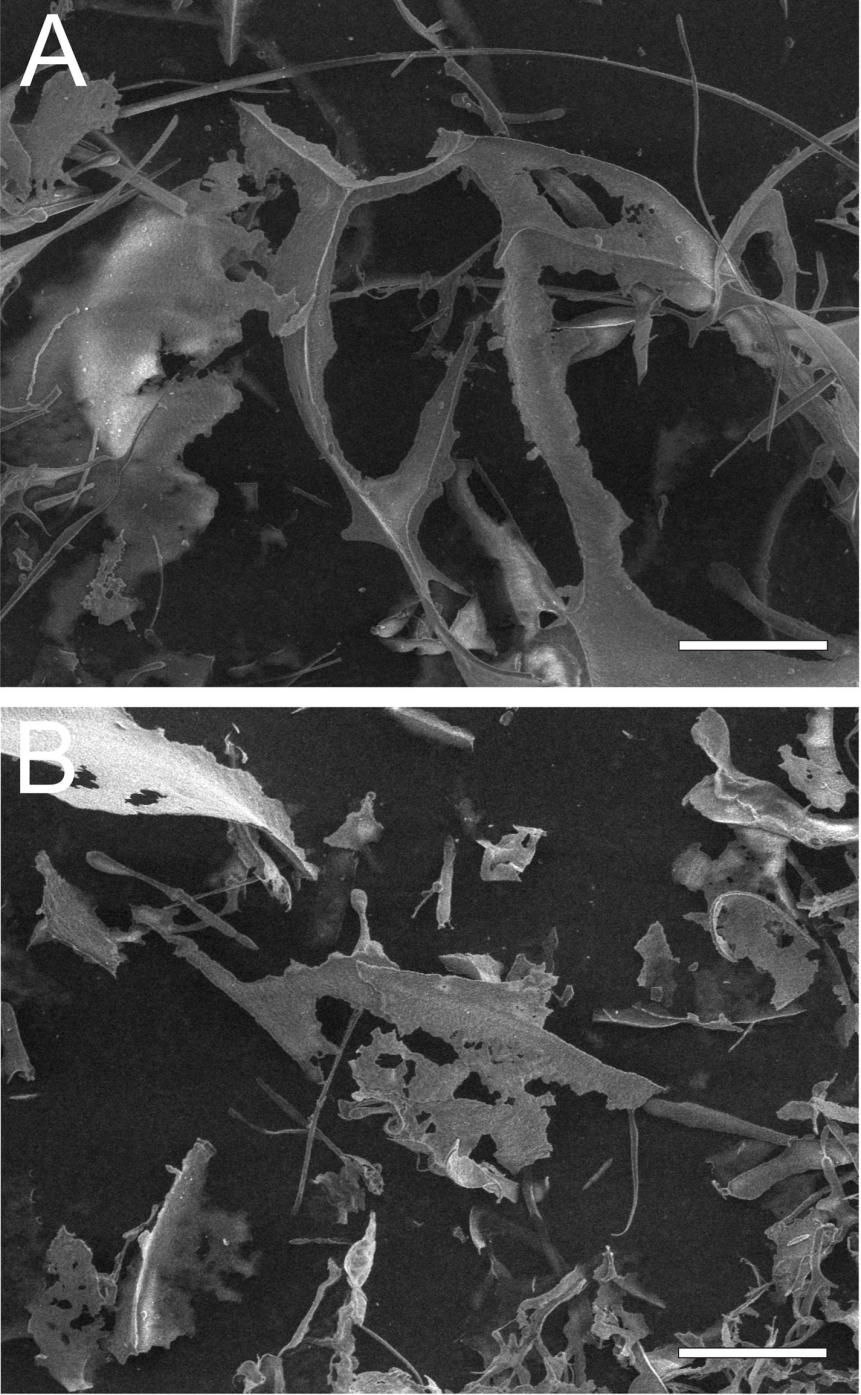
SEM image of the AAF. Fraction lyophilizate documented using Quanta^TM^ 3D FEG. Bars represent 50 μm.

The SEM analysis of the AAF showed the presence of large irregular structures reaching up to 100 nm and even larger (Fig 5A and 5B). These objects resembled plaques with jagged edges. Since all structures looked similar and no distinctly different morphological forms were observed, the fraction was analyzed with the FTIR and Raman spectroscopy techniques.

### FTIR analysis of the AAF

**Fig 6:**
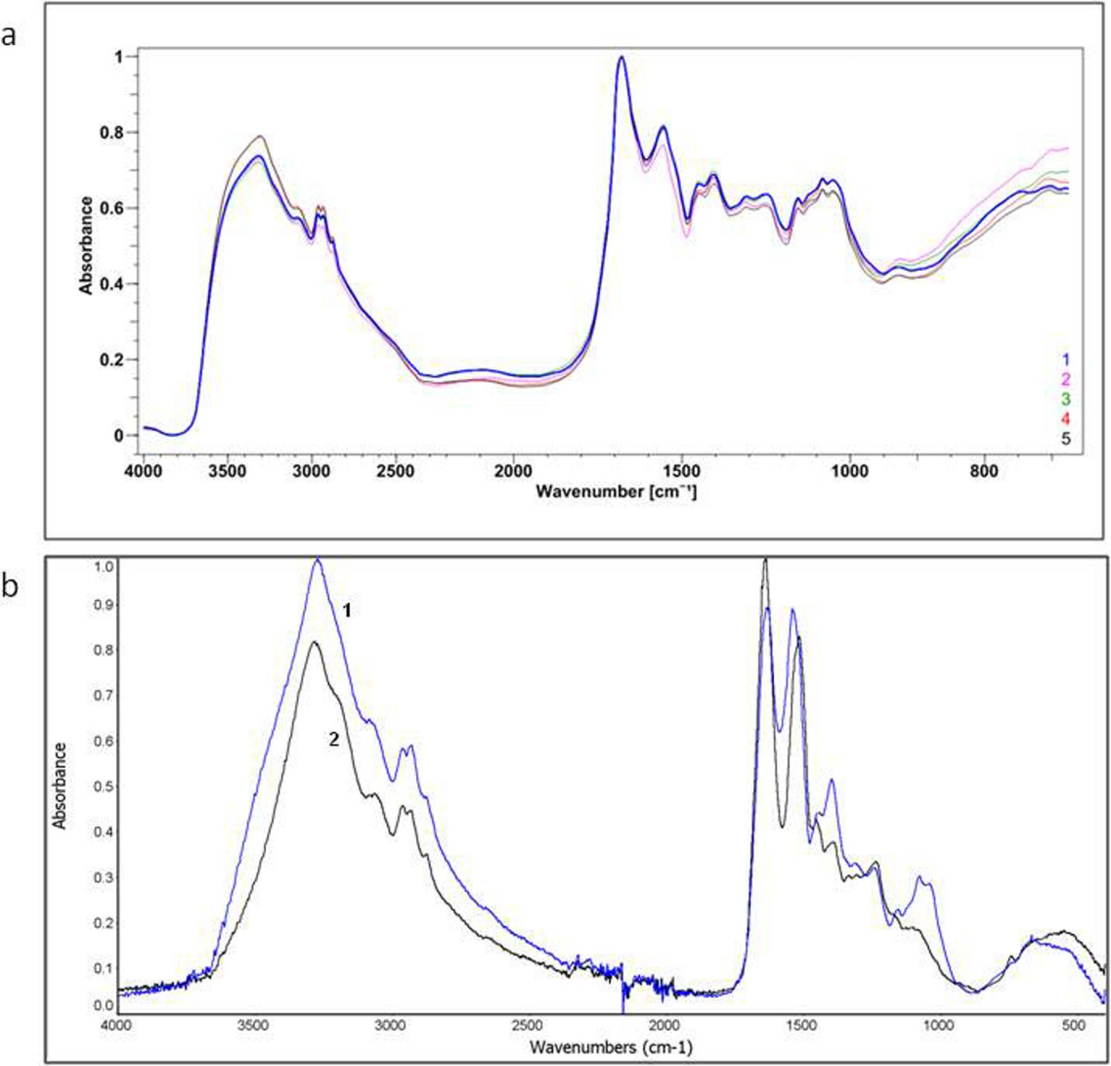
**a)** FTIR spectrum of the AAF. Spectra obtained from the 5 analyzed areas (50 x 50 μm). Each spectrum marked with a different color represents another analyzed area. **b)** ATR—FTIR spectrum of the AAF–(1) and egg white lysozyme–(2). The AAF spectrum showed 82% similarity to the spectrum of egg white lysozyme.

The lyophilizate of the AAF was observed under a FTIR microscope and the surfaces of several areas to be scanned were selected for the FTIR analysis. The analyzed areas did not differ between each other; this indicates chemical homogeneity of the analyzed material (Fig 6). The spectra of five different areas were compiled and marked with different colors in Fig 6A.

The ATR-FTIR spectrum of the AAF showed high similarity (82%) to the spectrum of the egg white lysozyme. The differences in the vibrations in the range of 1040–1156 cm^-1^ indicated the presence of polysaccharides in the AAF. The maximum absorbance for all polysaccharides is found between 950–1200 cm^-1^, where the COC and COH link band positions are identified [39]. Absorption bands in the range 1040–1070 cm^-1^ indicated the carbohydrate backbone and 1152–1156 cm^-1^—the glycosidic linkage in the saccharide structure [40] (Fig 6B).

### Lysozyme activity of the AAF

The lysozyme-type enzyme activity was compared in an unheated fraction (above 14 kDa) and after heating for 10 minutes at 70°C. The activity of the unheated fraction was on average 7072 U/mg (5480–8655 U/mg). The lysozyme-type activity of the AAF fraction was on average 2426 U/mg (764–3450 U/mg). The average value was determined based on measurements of 10 different series of this preparation.

### Polyacrylamide gel electrophoresis analysis of the AAF

**Fig 7:**
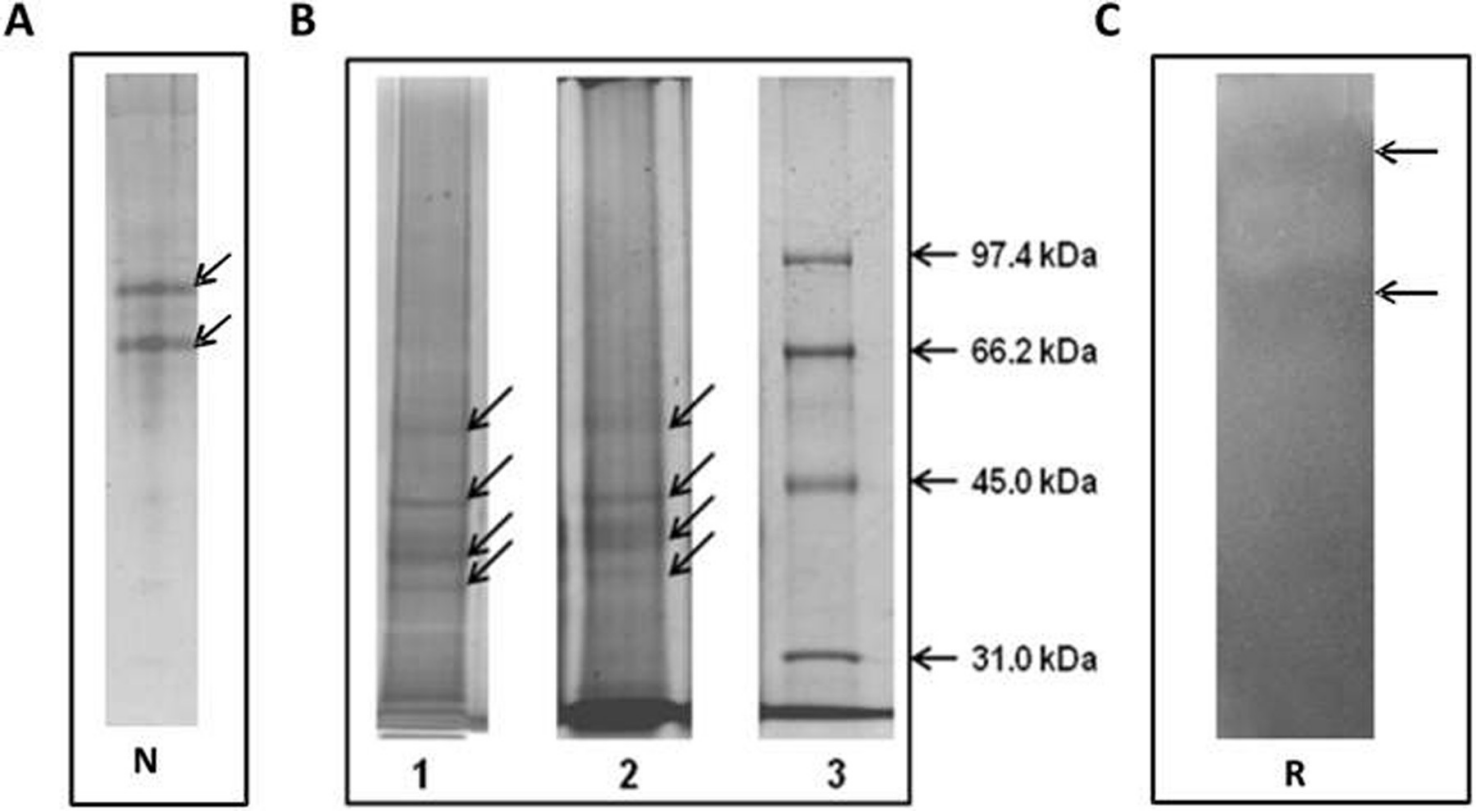
Electrophoretic analysis of the AAF, A—native electrophoresis (N); B—SDS/ PAGE electrophoresis: line 1—compounds stained with silver nitrate, line 2—protein bands stained with Coomassie Brilliant Blue R-250 (Sigma), line 3—molecular weight markers (Bio-Rad). The analyses were performed in 10% polyacrylamide gels. Stained bands are indicated by arrows. C—Detection of lysozyme-like activity by bioautography (Reisfeld—R method) after incubation for 48 h at 30°C. The analyses were performed on 50 μg of protein in the samples in 15% polyacrylamide gels. The arrows indicate the lytic zones of *M*. *luteus*.

Two bands were observed on the gel in the analysis of the AAF performed using electrophoresis under native conditions and staining to detect protein compounds (Fig 7A). After analyzing the fractions in denaturing conditions, five distinct protein bands were detected (Fig 7B line 2). After staining the gel for detection of sugars, the bands were stained at the same points on the gel (Fig 7B, line 1). This suggests that the analyzed compounds are protein-sugar compounds.

The AAF was analyzed using the bioautography technique after electrophoretic separation of native proteins in acidic polyacrylamide gels. Two lytic zones of *M*. *luteus* indicating lysozyme-type activity in the AAF were observed after incubation for 48 hours at 30°C (Fig 7C). The zones corresponded to the bands on the gel after native electrophoresis shown in Fig 7A.

### MALDI analysis of the AAF

#### Intact analysis

**Fig 8:**
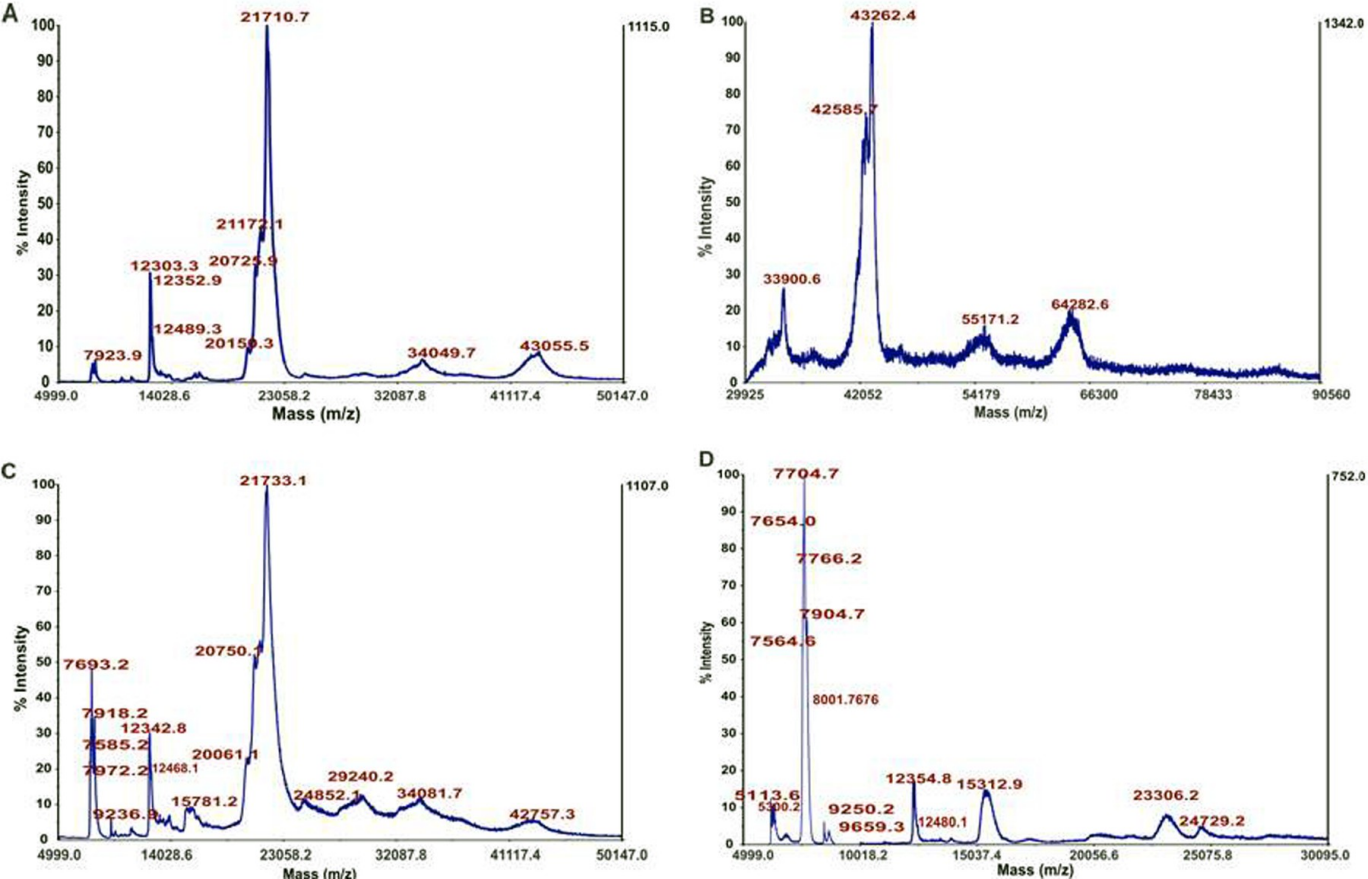
MALDI MS analysis of the intact spectra of the AAF: A) linear middle mass mode SA matrix, B) linear high mass mode SA matrix, C) linear middle mass mode sDHB matrix, and D) linear middle mass mode DHB matrix.

One of the techniques employed to obtain information about the protein composition of the AAF is mass spectrometry (MS). Especially useful is the matrix-assisted laser desorption ionization (MALDI) technique, which is quite resistant to impurities, and the AAF or lysates can be analyzed without any preliminary fractionation. In this case, a registered intact spectrum can show the general protein profile of the analyzed sample, which can be further used for batch-to-batch comparison or sample fingerprinting. In this study, three different matrices were employed to get as much information as possible about the investigated AAF. Sinapinic acid (SA), which is mostly used in the positive ion mode, allows detection of high-weight molecules, e.g. proteins that are larger than 5000 Da. 2,5-dihydroxybezoic acid (DHB) detects low-weight molecules such as peptides, nucleotides, oligonucleotides, and oligosaccharides, which do not ionize well with SA in the positive ion mode. The last one is a binary matrix composed of DHB and 2-hydroxy-5-methoxybenzoic acid. It is the softest matrix, ensuring improvement of m/z signals of large proteins and glycoproteins. The resulting mass spectra are shown in Fig 8. All of them confirmed the proteinaceous character of the sample. In the case of the SA matrix, the samples were analyzed in two linear instrument modes: middle mass and high mass. As shown in Fig 8A, the middle mass spectra exhibit a limited number of sharp peaks in the m/z range between 5 and 22 kDa and broad and low-intensity peaks between 23 and 50 kDa. In all cases, they seem to consist of several proteins or protein-protein complexes. The analysis in the linear high mass mode revealed the presence of large proteins even at ca. 65 kDa. In the case of signals between 42 and 44 kDa, multiple maxima of the m/z peaks can be recognized, showing again the complex character of the analyzed fluid. In the case of the binary sDHB matrix, it is possible to find clear similarities with the protein pattern presented in sinapinic acid with broader peaks in the range from 20 to 50 kDa. Classical 2,5-dihydroxybenzoic acid revealed a different spectrum, where there is no 21 kDa m/z. The spectrum is dominated by m/z at approximately 7.7 kDa, and the remaining part is composed of low-intensity peaks.

#### LC-MS/MS analysis of the AAF

We employed a classical proteomic approach to identify proteins present in the AAF. First, the identification was performed on the AAF (see Materials and Methods), which was dissolved in 8 M urea and treated with dithiothreitol and iodoacetamide to reduce and alkylate the present disulfide bonds. Afterwards, the sample was divided into three parts and digested by trypsin, chymotrypsin, and Lys-C, respectively. All samples were prepared according to the standard protocol (see Materials and Methods) for LC-MS/MS analysis. Because the number of proteins for *D*. *veneta* in the Uniprot database is quite poor and contains mostly sequences of cytochrome C, a higher database, Annelida, was chosen as a starting point. We were able to identify 7 proteins (Table 1), where the numbers of confident protein identifications were proven by detection of at least 2 tryptic peptides with peptide confidence of at least 95%.

**Table 1:** List of all proteins identified after tryptic digestion of the AAF (protein FDR<1%) along with their occurrences in different experiments mentioned by Unused ProtScores, numbers of peptides (95% confidence), total and percent of sequence coverage. The empty cells indicate lack of identification at the selected protein FDR threshold in a given experiment. The results of search against the Annelida database; and against the Lophotrochozoa database.

**Trypsin Annelida**							
**N**	**Unused**	**Total**	**% Cov**	**Accession #**	**Name**	**Species**	**Peptides(95%)**
1	48,14	48,14	98,7	O18423	Lysenin	EISFE	60
2	30,19	30,19	86,2	P92176	Actin-2	LUMTE	26
3	29,4	44,32	96,3	O18425	Lysenin-related protein 2	EISFE	106
4	8,66	8,66	71,5	P13579	Extracellular globin-4	LUMTE	13
5	8,08	8,08	77,9	Q7JQD3	Gelsolin-like protein 1	LUMTE	8
6	4	4	57,2	P02218	Extracellular globin-2	LUMTE	4
7	2,06	7,12	60,3	O18424	Lysenin-related protein 1	EISFE	9
8	2	2	80,7	Q6QLW4	Cytochrome c	PECGU	1
9	2	2	67,8	P21523	Homeobox protein LOX2 (Fragment)	HIRME	1
10	2	2	47	O77303	40S ribosomal protein S13	LUMRU	1
11	2	2	82,5	P84589	Ubiquitin (Fragment)	LUMTE	1
12	1,54	1,54	60	P02217	Globin, minor monomeric component (Fragment)	GLYDI	1
13	0,92	0,92	70,5	P30754	Fibril-forming collagen alpha chain (Fragment)	RIFPA	1
14	0,48	0,48	62,3	Q8MPM1	Gelsolin-like protein 2	LUMTE	2
15	0,27	0,27	56,4	O77302	40S ribosomal protein S10	LUMRU	1
**Trypsin Lophotrochozoa**							
**N**	**Unused**	**Total**	**% Cov**	**Accession #**	**Name**	**Species**	**Peptides(95%)**
1	38,79	38,79	87,9	O18423	Lysenin	EISFE	56
2	26,95	34,28	82,3	O18425	Lysenin-related protein 2	EISFE	101
3	20,83	20,83	67,3	P92176	Actin-2	LUMTE	18
4	12,21	12,21	27,5	A0A088BZ83	SCBP2-like protein (Fragment)	EISFE	19
5	8,06	8,06	46,4	P13579	Extracellular globin-4	LUMTE	12
6	8,03	8,03	45,5	Q7JQD3	Gelsolin-like protein 1	LUMTE	7
7	7,49	7,49	27,5	T1FNE3	Tubulin alpha chain	HELRO	4
8	4,86	4,86	26,5	V4AVP5	Tubulin beta chain	LOTGI	3
9	3,24	3,24	16,6	P02218	Extracellular globin-2	LUMTE	3
10	2,01	2,01	24,5	T1FNZ9	Uncharacterized protein	HELRO	1
11	2	4,86	25,9	A4GVQ1	Tubulin beta chain (Fragment)	ENCBU	4
12	2	2,01	19,9	A0A0L8FRZ6	Uncharacterized protein	OCTBM	1
13	2	2	23,8	B7SHS5	Coactosin-like protein	EISFE	2
14	2	2	31,6	E9NPR7	Glutathione S-transferase	EISFE	1
15	2	2	16,7	J7FBH7	Ubiquitin/ribosomal protein S27	CRAGI	1
16	1,6	1,6	15,1	Q5CD25	Valosin containing protein-1	EISFE	1
17	0,39	0,39	24,5	Q2I6A4	Superoxide dismutase (Fragment)	EISFE	2
18	0,07	0,07	14,2	A0A1S3K9V2	ubiquitin-conjugating enzyme E2 L3-like	LINUN	0
19	0,06	0,06	30,4	A0A088BZM2	Neurocalcin-like protein (Fragment)	EISFE	0
20	0,05	0,05	7,6	K1PW06	Filamin-C	CRAGI	1

The highest sequence coverage with a high number of identified peptides confirmed the presence of lysenin (sequence coverage up to 98%, organism: *Eisenia fetida*), actin-2 (sequence coverage up to 86%, organism: *Lumbricus terrestris*), and lysenin-related protein 2 (sequence coverage up to 96%, organism: *E*. *fetida*). To detect the presence of other possible proteins, we searched the results against Lophotrochozoa, and the outcome confirmed the presence of most of the proteins identified in the first search, but also revealed additional proteins. In this case, we were able to identify the same three main components: lysenin, lysenine-related protein 2, and actin-2. In addition to these three proteins, the presence of a calcium ion binding SCBP2-like protein fragment (sequence coverage up to 45%) also present in *E*. *fetida* is worth noting.

To get complete proteomic coverage, two enzymes alternative to trypsin were used: chymotrypsin and endoproteinase Lys-C. Table 2 presents proteins identified after complementary digestion of the AFF fraction. Searching the results against the Annelida and Lophotrochozoa databases confirmed the presence of three major proteins identified previously based on tryptic peptides.

**Table 2:** List of all proteins identified after Lys-C and chymotryptic digestion of the AAF (protein FDR<1%) along with their occurrences in different experiments mentioned by Unused ProtScores, numbers of peptides (95% confidence), total and percent of sequence coverage. The empty cells indicate lack of identification at the selected protein FDR threshold in a given experiment.

**LysC Annelida**										
**N**	**Unused**	**Total**	**% Cov**		**Accession #**		**Name**		**Species**	**Peptides(95%)**
1	62,03	62,03	77,7		O18425		Lysenin-related protein 2		EISFE	65
2	28,13	46,05	72,1		O18423		Lysenin		EISFE	37
3	20,05	20,05	43,4		P92176		Actin-2		LUMTE	10
4	4	6,02	46,3		O18424		Lysenin-related protein 1		EISFE	7
5	2	8,02	39,7		Q3LX99		Lysenin-related protein 3		EISFE	8
6	2	2	19,5		Q9GRJ1		Calmodulin		LUMRU	1
7	2	2	6,9		P02218		Extracellular globin-2		LUMTE	1
8	0,16	0,16	17,5		O15991		Lombricine kinase		EISFE	1
9	0,08	0,08	7,4		P84239		REVERSED Histone H3		URECA	0
**LysC Lophotrochozoa**										
**N**	**Unused**	**Total**	**% Cov**		**Accession #**		**Name**		**Species**	**Peptides(95%)**
1	56,07	56,07	77,7		O18425		Lysenin-related protein 2		EISFE	61
2	30	50	71		O18423		Lysenin		EISFE	36
3	20	20	36,7		P92176		Actin-2		LUMTE	10
4	8	8	50,8		A0A088BZ83		SCBP2-like protein (Fragment)		EISFE	6
5	4,55	8,68	39,7		O18424		Lysenin-related protein 1		EISFE	9
6	4	4	17,7		W5VJG5		Elongation factor 1-alpha-like isoform X1 (Fragment)		BIOGL	2
7	4	4	20,3		B7SHS5		Coactosin-like protein		EISFE	2
8	3,22	10,28	39		Q3LX99		Lysenin-related protein 3		EISFE	9
9	2	2	42,9		D0EP16		Pi-class glutathione S-transferase (Fragment)		EISFE	1
10	2	2	13,3		K1PZ90		Uncharacterized protein		CRAGI	1
11	2	2	5		A0A210QMJ2		Myeloid differentiation primary response protein MyD88		MIZYE	2
12	2	2	7		A0A0M3KKX6		Extracellular globin		9ANNE	1
13	2	2	7,3		A0A0L8FRZ6		Uncharacterized protein		OCTBM	1
14	0,3	0,3	3,8		A0A0B7B860		REVERSED Uncharacterized protein		9EUPU	1
**Chymotrypsin Annelida**										
**N**	**Unused**	**Total**	**% Cov**		**Accession #**		**Name**		**Species**	**Peptides(95%)**
1	59,84	59,84	92,9		O18423		Lysenin		EISFE	41
2	23,58	41,47	86,7		O18425		Lysenin-related protein 2		EISFE	42
3	13,42	13,42	77,1		P92176		Actin-2		LUMTE	11
4	4,51	4,51	49,1		Q7JQD3		Gelsolin-like protein 1		LUMTE	3
5	0,64	0,64	59,6		P13579		Extracellular globin-4		LUMTE	0
6	0,28	0,28	86,1		Q34949		REVERSED NADH-ubiquinone oxidoreductase chain 4		LUMTE	0
7	0,21	2,87	74,7		Q3LX99		Lysenin-related protein 3		EISFE	3
8	0,05	0,05	75,2		Q34949		NADH-ubiquinone oxidoreductase chain 4		LUMTE	0
**Chymotrypsin Lophotrochozoa**										
**N**	**Unused**	**Total**	**% Cov**		**Accession #**		**Name**		**Species**	**Peptides(95%)**
1	58,31	58,31	86,9		O18423		Lysenin		EISFE	45
2	30,79	45,92	80		O18425		Lysenin-related protein 2		EISFE	43
3	16,37	16,37	55,6		P92176		Actin-2		LUMTE	14
4	4,22	4,22	50		A0A088BZ83		SCBP2-like protein (Fragment)		EISFE	2
5	0,67	0,67	29,4		T1G071		Uncharacterized protein		HELRO	1
6	0,2	0,2	17,1		A0A0B7BQZ1		REVERSED Uncharacterized protein (Fragment)		9EUPU	0

#### Gel analysis of the AAF

To obtain more information about the AAF composition, 2D gel analysis was performed (Fig 7B). The most intensive bands present at around 55 kDa, 45 kDa, 42 kDa, and 35 kDa were cut and prepared for the LC-MS/MS analysis (see the Materials and Methods section). The data obtained were searched against Annelida and Lophotrochozoa databases (Uniprot), and the results are presented in S1 Table in Supporting Information. Surprisingly, the comparison of the proteins identified with the former database showed the same composition of all the four gel bands: lysenin-related protein 2, actin-2, and lysenin. The analysis of the protein list obtained after the search against Lophotrochozoa revealed differences between the bands. The highest band cut around 55 kDa except lysenin and lysenin-related protein 2 contained many different tubulin chains. The second band (~ 45 kDa) presents different additional actins. The last two bands are mostly composed of basic proteins (lysenins) with minor addition of tubulins, actins, and an SCBP2-like protein fragment.

### Raman spectroscopy analysis of the AAF proteins

**Fig 9:**
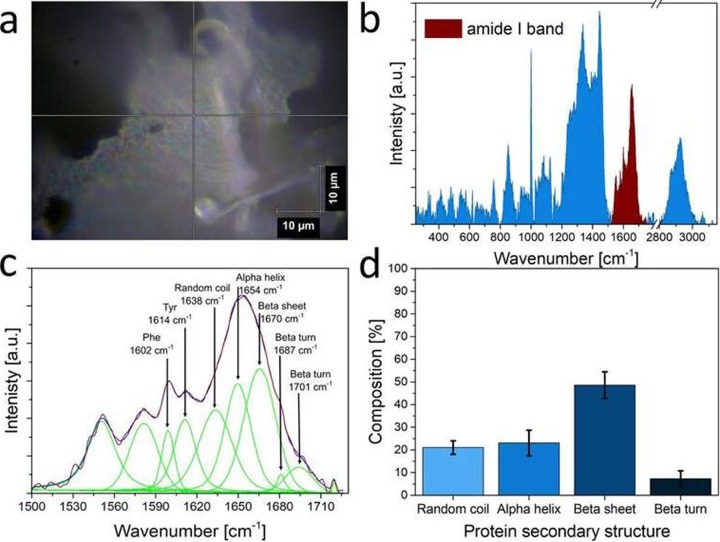
Example of the measurement site of the Raman spectrum collected on the surface of the AAF (a). Raman spectrum of the AAF with the amide I band selected for the analysis (b). Example of the deconvolution of the amide I band (c). Percentage content of the secondary structure of the particular proteins in the AAF (d).

Raman spectroscopy was applied to identify the secondary structure of the AAF proteins. It is an effective tool for analysis of the composition and type of proteins [41–44]. Moreover, this method distinguishes the type of the secondary structure in proteins (alpha-helix, beta-sheet, or beta-turn) [41, 42, 45–48]. Fig 9 shows an example of a Raman spectrum of the proteins in the AAF (b) and the site of measurement on the material surface (a). The amide I band in the Raman spectrum, which was used in the analysis of the AAF protein secondary structure, is labeled. To obtain the percentage content of the type of the protein secondary structure, the curve-fitting process of the amide I band was used to distinguish overlapping bands. The curve-fitting process in the amide I band region of the Raman spectrum is presented in Fig 9C. The bands assigned to the alpha-helix and beta-sheet conformations are visible at 1654 cm^-1^ and 1670 cm^-1^, respectively. The band associated with the random coil structure is detected at 1638 cm^-1^, whereas the bands representing the beta-turn conformation are found at 1687 cm^-1^and 1701 cm^-1^. Moreover, the bands at 1602 cm^-1^ and 1614 cm^-1^ are assigned to phenylalanine (Phe) and tyrosine (Tyr) ring modes, respectively. The integrated intensity of the bands in the amide I region gives reasonably accurate estimates for the alpha-helix, beta-sheet, beta-turn, and random coil content in AAF proteins. Fig 9D shows the percentage content of particular protein secondary structures in the studied material obtained by the analysis of the band intensity in the amide I region.

### NMR analysis of the CF fraction

**Fig 10:**
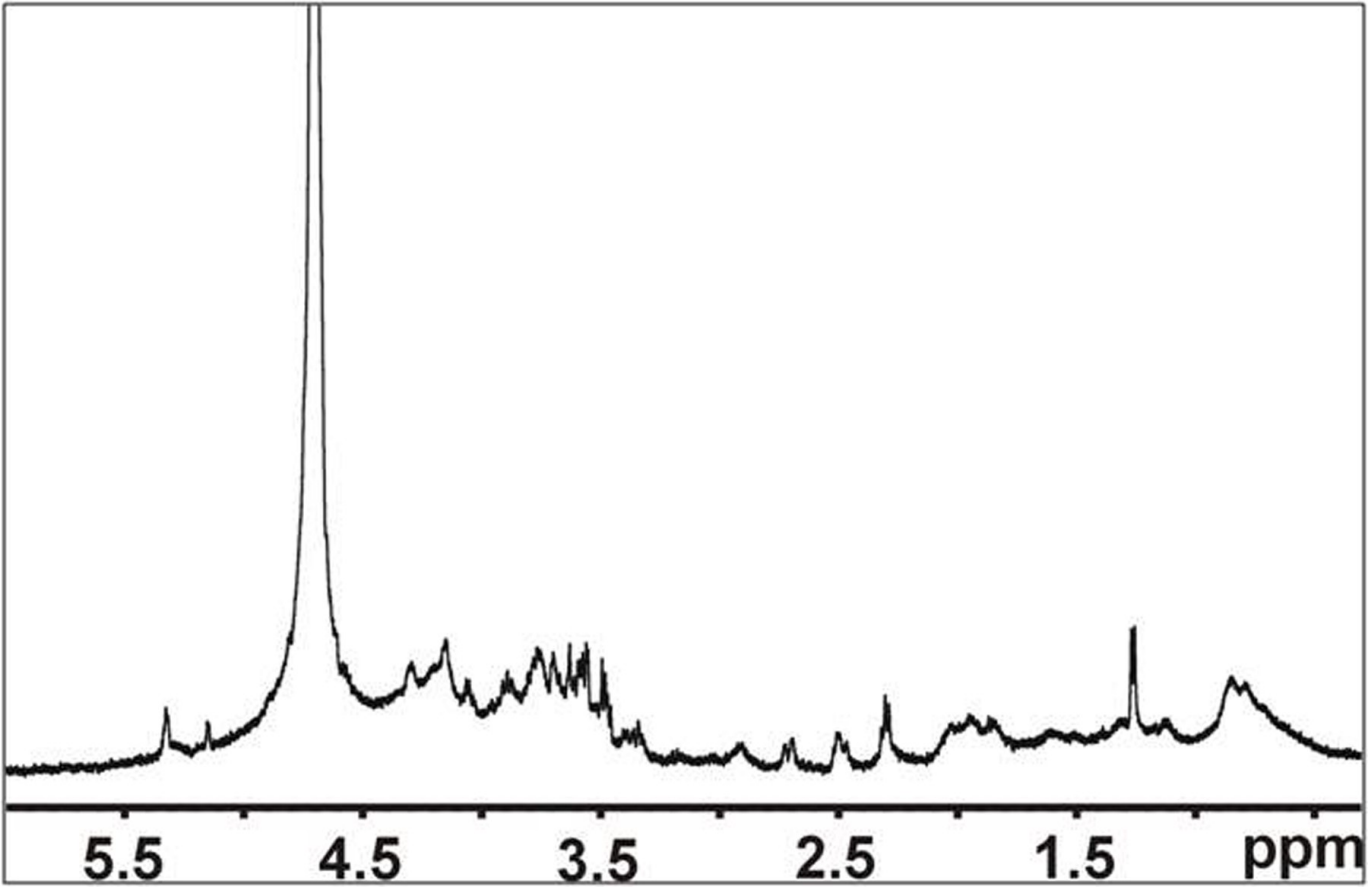
^1^H NMR spectrum of the AAF. Signals in the 5.1–5.4 ppm and 3.4–4.4 ppm regions are characteristic for carbohydrates.

The ^1^H NMR spectrum of the AAF contains signals in the 5.1–5.4 ppm and 3.4–4.4 ppm regions, which are characteristic for carbohydrates (Fig 10). Moreover, the broad shape of the signals confirmed the high molecular weight of this fraction.

### Composition of carbohydrates

Inositol, glucose, galactose, and N-acetylglucosamine carbohydrates in a molar ratio of 18.5:14.8:51.8:14.9 were identified in the AAF samples subjected to strong acid hydrolysis.

### Determination of the cytotoxic activity of the AAF

**Fig 11:**
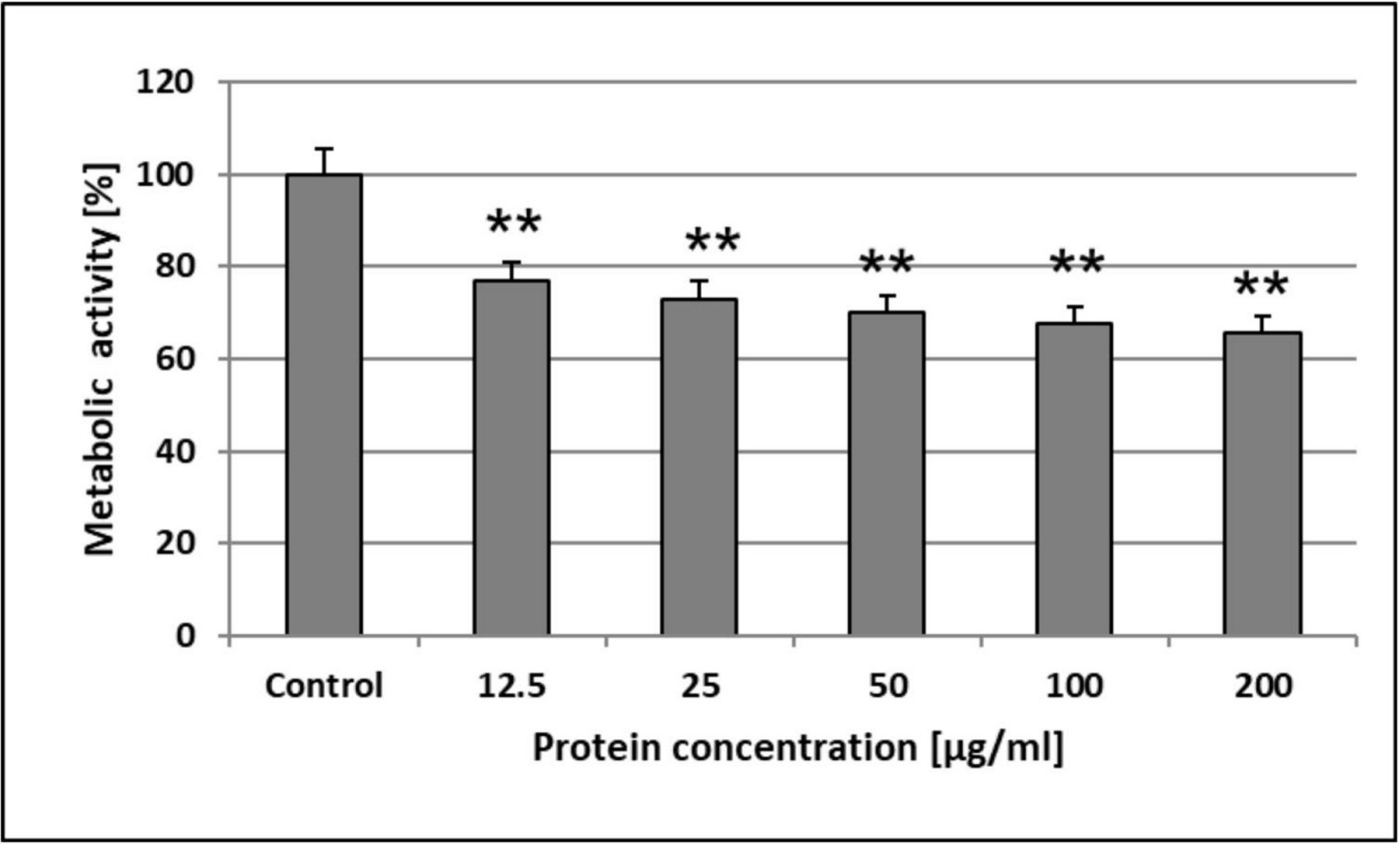
The AAF impact on the metabolic activity of HSF cells. The results were obtained from 3 independent experiments; *P<0.01 compared to the control group.

The MTT analysis revealed that the AAF decreased the metabolic activity of HSF cells in a concentration-dependent manner. We showed that the lowest concentration applied (12.5 μg mL^-1^) significantly decreased succinate dehydrogenase activity to 23.3%, in comparison to the unstimulated control. The increase in the AAF concentration (up to 200 μg mL^-1^) caused limitation of the enzyme activity by another 10.9% only (Fig 11). This may suggest that the AAF exerts its activity mainly at lower concentrations.

The AgNORs analysis showed no significant changes in the size and number of nucleolar organizer regions, suggesting a very limited impact of the tested AAF on the proliferative activity of normal skin fibroblasts. Given these results, we can suggest that the tested compound exerts only a cytostatic effect on HSF cells, and in our opinion, cannot be considered as a factor destroying the normal skin layer and subcutaneous tissue.

## Endotoxin content in the AAF

Detection and quantitation of endotoxin in the AAF was conducted using the standard LAL test. The endotoxin content in the AAF determined with the LAL assay was low, i.e. 0.1 EU mL^-1^, corresponding to approx. 10 pg mL^-1^. This is lower than the endotoxin limit for transdermally administered substances set at 3.33 EU mL^-1^ according to endotoxin limits for parenteral drug products [49].

## Effect of temperature and pH on the anti-*C*. *albicans* activity of the AAF

**Fig 12:**
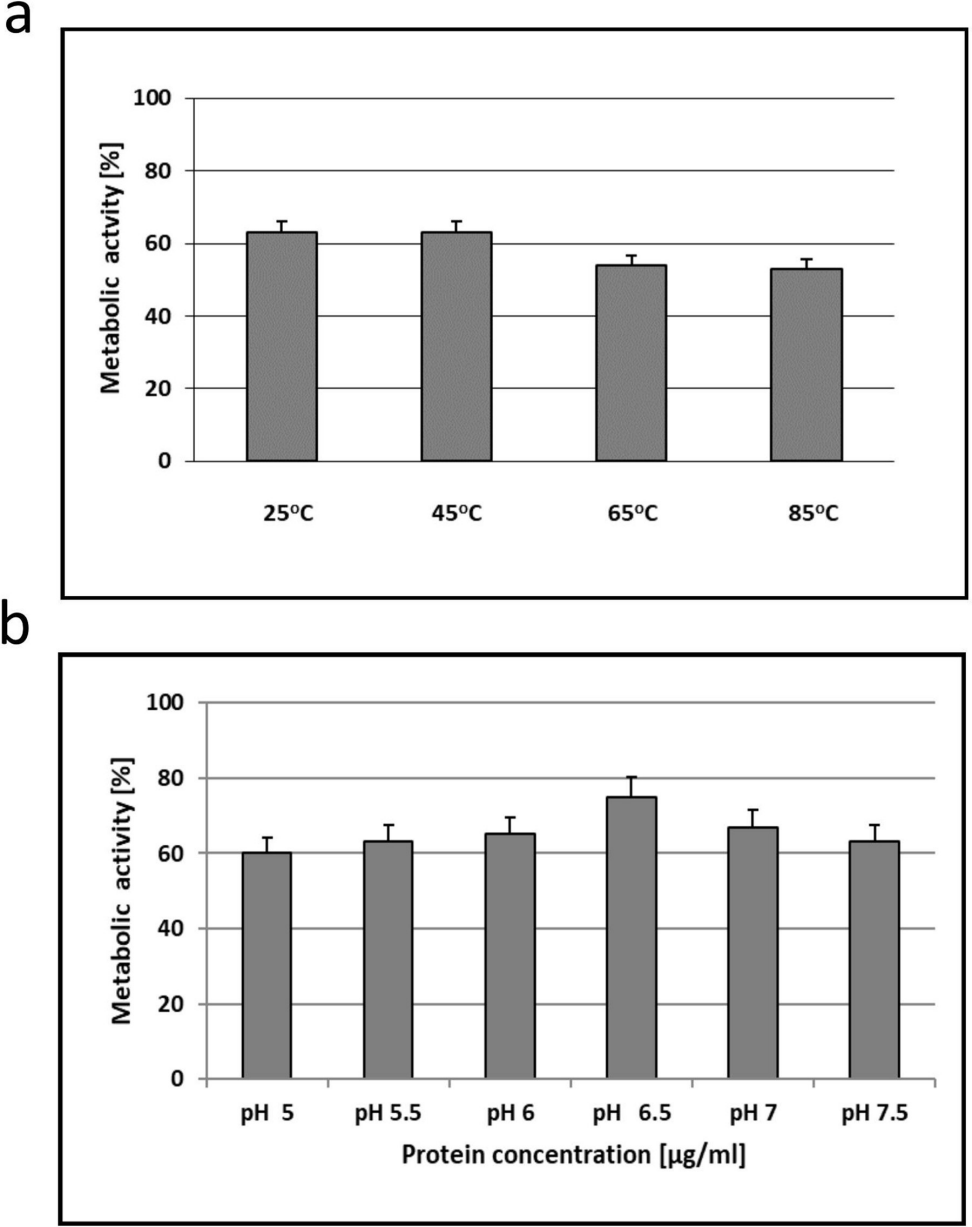
Effect of temperature **a)** and pH **b)** on the anti-*C*. *albicans* metabolic activity of the AAF.

After the incubation of the AAF in the water solution (at the protein concentration of 100 μg mL^-1^) at 25°C and 45°C for 10 minutes, the metabolic activity of *C*. *albicans* ATCC 10231 was at a same level of 63%. However, after the incubation at 65°C and 85°C, it decreased by 10% and was similar as well (Fig 12A).

The pH can affect the rate and degree of dissolution of the AAF. The metabolic activity of fungal cells increased after dissolving the AAF in Sörensen buffer at pH from 5.0 to 6.5 and then decreased at pH 7 and 7.5, but the differences were not statistically significant (Fig 12A).

## Discussion

Most investigations are focused on isolation and identification of active substances from biological preparations. However, the compounds are not always applicable due to their insufficient activity or cytotoxicity to normal cells. In plant and animal extracts, bioactive compounds usually act synergistically. Another way is to find an active extract or fraction showing high biological activity and no cytotoxicity towards normal cells and then identify its components. These studies were carried out with this aim.

In the present study, the *D*. *veneta* CF fraction with activity against *C*. *albicans* was obtained and characterized for the first time. The AAF effectively destroyed the cells of the clinical *C*. *albicans* isolate and showed activity against *C*. *albicans* ATCC 10231 and non- albicans strain *C*. *krusei* ATCC 6258. The AAF caused destruction of the fungal cell wall and both apoptotic and necrotic death. The physicochemical conditions such as pH and temperature of the AAF solution did not significantly affect the metabolic activity of *C*. *albicans* cells.

The analysis of the AAF proved the presence of protein and sugar compounds in this fraction. The Raman analysis confirmed the occurrence of proteins and determined their secondary structure, while the MALDI TOF/TOF analysis indicated the presence of four main compounds with a mass of 7694.9 m/z, 12292.3 m/z, 21628.3 m/z, and 42923.2 m/z. The FTIR analysis showed that, after lyophilization, the fraction was chemically homogeneous.

The preparation was divided into three subfractions in terms of molecular weight. Each of the three separated subfractions (14–30 kDa, 30–100 kDa, and above 100 kDa) exhibited weaker antifungal activity than the AAF. While the isolated subfractions (at 100 μg mL^-1^) reduced the *C*. *albicans* metabolic activity by 24%, 30%, and 40% respectively, the AAF fraction reduced this activity by as much as 88%. This indicates synergistic action of the compounds contained in the AAF.

The mass spectrometry analysis of the biologically active fraction of *D*. *veneta* CF revealed the protein composition of the AAF. The digestion with three proteolytic enzymes clearly showed that the main protein core consisted of lysenin and lysenin-related protein 2. This group of proteins is well characterized in earthworms, for example in *E*. *fetida*. In the Uniprot database, there is no entry for lysenins from *D*. *veneta*. In our study, proteins were identified based on the sequences described for *E*. *fetida*, and the sequence coverage for the results obtained oscillated at a level from 77 to 98%. The molecular masses of lysenins vary between 29 and 35 kDa, and the MALDI fingerprint of the AAF showed broad m/z signals in this range. Another main component is actin-2 (*L*. *terrestris*, MW 41.8 kDa), which can be observed on MALDI MS as a quite narrow signal at m/z around 42–43 kDa. The search of the MS/MS results against the Lophotrochozoa database revealed the presence of a fragment of soluble calcium-binding proteins 1 (SCBP1, *E*. *fetida*, MW 13.8 kDa). Since the sequence cover achieves nearly 50 percent in this case and, hence, the sequence compliance may be lower, the only m/z value present in the MALDI spectrum that can be assigned to this protein is the signal at 12300 m/z (Fig 8A, 8C and 8D). At m/z around 15000, we can probably observe extracellular globin 1 and 2 (Fig 8C and 8D) identified in the proteomic analysis as well. In the group of the identified proteins, only lysenins exhibit antibiotic, antibacterial, and toxic properties; therefore, they can be regarded as one of the components responsible for the toxic activity against *C*. *albicans*.

The lack of cytotoxicity of the preparation against normal human cells is a key factor for qualification thereof for further study, as the AAF did not show cytotoxicity against human skin fibroblasts. The ESI-LC MS/MS analysis of the five main bands identified after electrophoresis in denaturing conditions revealed the presence of fragments with sequences showing high similarity to the lysenine sequence (75–87%). It can be assumed that these fragments originated from the denaturation of this protein. At the same time, the analyzed preparation lost its cytotoxic activity. Lysenin, i.e. a pore-forming toxin found in the earthworm *E*. *fetida*, specifically interacts with sphingomyelin. It plays an important role in innate immunity against parasites by attacking their membranes and forming pores [50]. Lysenin is an oligomer and should be further investigated to check whether the detected lysenin fragments play any role in the activity of the studied fraction.

Based on the results obtained from the proteomic analyses, however, we are not able to identify clearly the proteins visible on the MALDI spectrum at m/z 7700 and 21700. The mass about 7700 m/z corresponds to the mass of a peptide lumbricin with antifungal activity that has been found in earthworms. This antimicrobial peptide with anti-*C*. *albicans* activity was isolated from skin secretion of the earthworm *Pheretima guillelmi* [11] and from homogenates of *Lumbricus rubellus* [12]. Lumbricin from *L*. *rubellus* also showed activity against two other strains, i.e. *Cryptococcus neoformans* and *Saccharomyces cerevisiae*. However, since this peptide has not been identified in the proteomic experiment, further analysis is necessary to identify the protein/peptide present at this m/z.

The antimicrobial mechanism of the earthworm CF is associated with lysozyme-type proteins [51]. The analyzed AAF fraction also exhibited lysozyme-type activity causing lysis of *M*. *luteus* cells. However, we were unable to identify lysozyme-like proteins based on the proteomic analysis. The compounds on the MALDI spectra at 12292.2832 m/z and 21628.2734 m/z, which were previously not assigned to any protein, may be considered as different isoforms of a lysozyme-like protein, but this has to be further investigated.

Fiołka and collaborators [51] observed previously that the antibody directed against human lysozyme recognized a protein of 22 kDa found in the CF of *D*. *veneta*. After electrophoretic analysis of native proteins of the CF in acidic polyacrylamide gels and using the bioautography technique, two lytic zones of *M*. *luteus* were observed. In the present study, two lytic zones of *M*. *luteus* were also found after analysis of the AAF using the same technique. This indicates the presence of two isoforms of lysozyme-like proteins both in the AAF and in the CF.

To obtain the AAF, the CF was collected in 0.9% NaCl, filtered, and heated for 10 minutes at 70°C. In these conditions, it lost its toxic properties and exhibited strong antifungal activity against *C*. *albicans*, which was not found in the native CF. The incubation temperature of 70°C and the presence of NaCl were favorable conditions for the oligomerization of lysozyme. However, lysozyme has an ability to form complexes with different compounds such as sugars, peptides, and acids, which can modify their molecules and change their activity [52–54].

Recent research indicates that lectin-like lysozymes play an important role in the immune defense function in humans. Lysozyme has the ability to form complexes with disaccharides and tetrasaccharides, acquiring unique properties by an unknown glycan-guided mechanism [55]. Our results clearly indicate the presence of protein-sugar compounds in the studied fraction. The ATR-FTIR analysis showed high similarity (82%) of the AAF spectrum to the spectrum of egg white lysozyme; however, the comparison of these spectra shows that the differences were mainly related to vibrations in the ranges characteristic for polysaccharides. The NMR analysis indicated the presence of sugars and the GC-MS analysis showed compounds such as inositol, glucose, galactose, and N-acetylglucosamine in the AAF.

Modern research confirms earlier observations regarding the agglutinative properties of lysozyme [56, 57]. In the earthworm *D*. *veneta*, a glyco-protein complex with activity against *C*. *albicans* was found. Metabolites of gut bacteria *R*. *ornithinolytica* isolated from this earthworm characterized as a carbohydrate-protein complex were found to exert a potent effect against *C*. *albicans*. The isolated complex also effectively reduced the metabolic activity of *C*. *albicans* cells. It caused cell agglutination and similar morphological changes in the fungal cells; however, cell death occurred only via necrosis [14, 15]. No apoptotic cells were observed in the study.

In conclusion, a protein–carbohydrate fraction that effectively destroyed *C*. *albicans* and *C*. *krusei* cells was obtained from the coelomic fluid of the earthworm *D*. *veneta*. Taking into account its effective action against *Candida* in the absence of endotoxicity and cytoxicity against normal skin fibroblasts, the AAF is a promising preparation with antifungal activity. Since the AAF showed high activity against the clinical *C*. *albicans* isolate, it is reasonable to continue further research with a view to application thereof in medicine.

## Supporting information

S1 Table List of all proteins identified for two databases (Annelida and Lophotrohozoa), in the gel bands (protein FDR<1%) along with their occurrences provided by Unused ProtScores, numbers of peptides (95% confidence), total and percent of sequence coverage. The empty cells indicate lack of identification at the selected protein FDR threshold in a given experiment.
(TIF)
