# Supplementary figures and images for: Anti-Candida albicans effect of the protein-carbohydrate fraction obtained from the coelomic fluid of earthworm Dendrobaena veneta
Source: PLoS One. 2019 Mar 11;14(3):e0212869. doi: 10.1371/journal.pone.0212869 (PMC6411149; doi:10.1371/journal.pone.0212869)

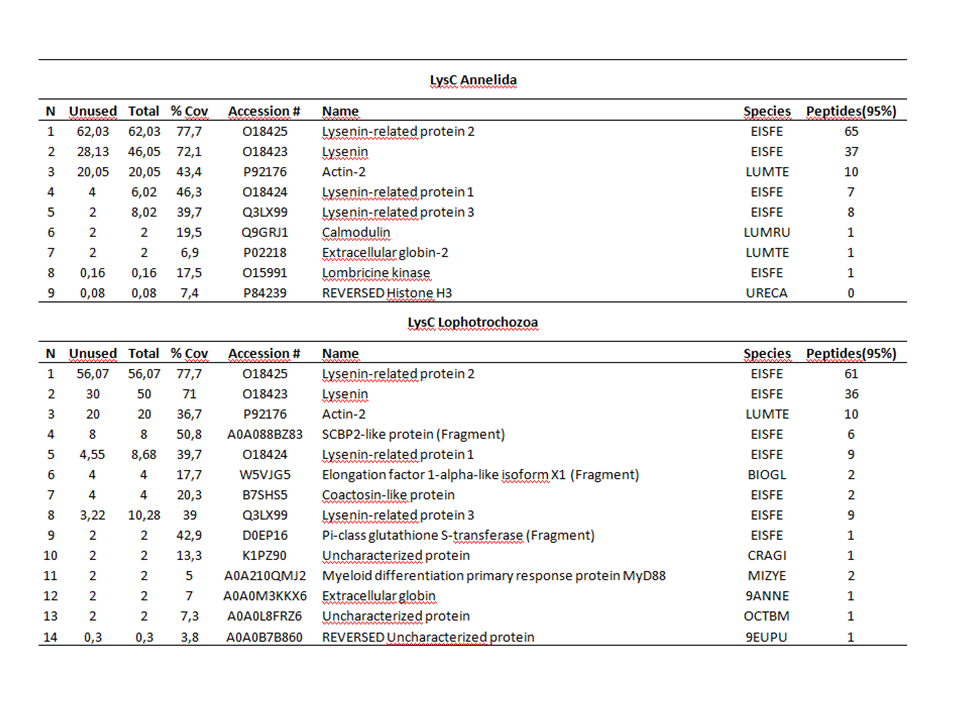

Supplement: S1 Table — The empty cells indicate lack of identification at the selected protein FDR threshold in a given experiment. (TIF) [file pone.0212869.s001.tif]
